# Pan-cancer analysis identifies tRNA modification enzyme CTU2 as a novel tumor biomarker and its role in immune microenvironment

**DOI:** 10.3389/fimmu.2025.1547794

**Published:** 2025-05-01

**Authors:** Jiaojiao Wang, Chang Gao, Junyi Zhang, Huahong Luo, Siqi Dai, Jianwei Wang

**Affiliations:** ^1^ Department of Surgery, The Fourth Affiliated Hospital of School of Medicine, International School of Medicine, International Institutes of Medicine, Zhejiang University, Yiwu, China; ^2^ Department of Colorectal Surgery and Oncology, Key Laboratory of Cancer Prevention and Intervention, Ministry of Education, Second Affiliated Hospital, Zhejiang University School of Medicine, Hangzhou, China

**Keywords:** CTU2, tRNA modification, pan-cancer, immune microenvironment, prognosis, USF1

## Abstract

**Background:**

Recent studies have highlighted dysregulated tRNA modifications in the reprogramming of tumor translation. Cytosolic thiouridylase subunit 2 (CTU2) is an essential and conserved enzyme that modifies tRNA at the wobble position. However, the relationship between CTU2 expression and various cancer types remains insufficiently explored.

**Methods:**

Pan-cancer data from TCGA, GEO, and CPTAC were used to analyze CTU2 expression and its prognostic value. Single-cell and spatial transcriptomic analyses were performed to identify CTU2’s cell-type labels and distribution. The TCGA microRNA database was used to explore the expression patterns of CTU2-modified tRNAs and their prognostic significance. TIMER2.0, ESTIMATE, and TIP were employed to analyze the correlation between CTU2 expression, immune infiltration, and immunotherapy response. GSEA and Depmap databases were conducted to explore signaling pathways related to CTU2 expression. Drug sensitivity related to CTU2 was assessed using CMap and GDSC-V2. The oncogenic roles of CTU2 were validated in vitro and in vivo. Genomic alterations, public ChIP-seq data, dual-luciferase assays, and EMSA were employed to investigate the upstream regulatory mechanisms regulating CTU2.

**Results:**

CTU2 and its modified tRNA, particularly tRNA-Lys-TTT, are differentially expressed across various tumor types, suggesting their potential as prognostic biomarkers. Abnormal CTU2 expression in tumors is associated with alterations in immune cell infiltration, immune evasion, and immunotherapy response. CTU2 may contribute to several key cancer-related pathways and biological processes. Mechanistically, CTU2 overexpression is likely driven by DNA copy number amplification and DNA methylation alterations. USF1 has been identified as one of the transcription factors regulating CTU2.

**Conclusions:**

CTU2 may serve as a valuable prognostic and immunotherapeutic biomarker across multiple cancer types, providing new insights into tumor treatment strategies and immune evasion from the perspective of tRNA modifications.

## 1 Introduction

tRNAs, once viewed as static adaptors transporting amino acids and interpreting mRNA codons (1, 2), are now recognized for their dynamic roles in regulating gene expression and translation (3–6). A recent study reveals that tRNAs act as ‘accomplices’ in dysregulated translation systems. Specifically, tRNA-Glu-TTC is significantly upregulated in highly invasive breast cancer cells, and its overexpression enhances the translation of mRNAs with complementary codons (GAA, which base-pair with TTC). This upregulation increases the translation efficiency of exosome component 2 (EXOSC2) and GRIP1-associated protein 1 (GRIPAP1), both of which are enriched in GAA codons within their coding regions, positioning them as key downstream mediators of the pro-metastatic effects of tRNA-Glu-TTC overexpression. These findings emphasize the role of codon-biased translation, driven by upregulated tRNAs, in promoting the synthesis of oncoproteins (7).

tRNA modifications are essential for proper tRNA folding, aminoacylation, stability, and mRNA decoding, ensuring optimized translation (8, 9). Recent studies have revealed that tRNA modifications can significantly influence the decoding capability of tRNA, promote its codon-biased translation, and play an active role in the dynamic regulation of gene expression (8, 10). Modifications in the tRNA anticodon loop are crucial for modulating tRNA decoding ability, as abnormal modifications directly affect the pairing between the tRNA anticodon and the mRNA codon (11, 12). CTU2 catalyzes the critical final 2-thiolation step necessary for the mcm^5^s^2^U cascade modification at the first position of the tRNA anticodon (position 34) in the anticodon loop of tRNAs (13). Notably, the first position of the tRNA anticodon, known as the wobble position, exhibits non-Watson-Crick base pairing with the third nucleotide of the codon. For instance, the unmodified base uridine (U) at the first anticodon site can pair not only with codon adenine (A) but also with guanine (G) and cytosine (C) (10, 14). This non-complementary pairing is relaxed and unstable, increasing the likelihood of frameshift errors during translation. In contrast, the 5-methoxycarbonylmethyl-2-thiouridine (mcm^5^s^2^U) modification strictly regulates and stabilizes the complementary base pairing between U and A, occurring exclusively in three specific tRNAs (tRNA-Glu-TTC, tRNA-Lys-TTT, and tRNA-Gln-TTG), where the 34th position is U (in the DNA sequence, this corresponds to thymine, T) (13, 15, 16). While wobble pairing expands the decoding capacity of tRNAs, the mcm^5^s^2^U modification restricts strict complementary pairing between the anticodon (TTC, TTT, TTG) and their corresponding U34 codons (GAA, AAA, and CAA) (13, 17). Thus, CTU2-mediated mcm^5^s^2^U modification is crucial for maintaining the accuracy and fidelity of translation.

CTU2-mediated mcm^5^s^2^U modification is crucial for maintaining the accuracy and fidelity of translation across various organisms (13, 18–20). In the nematode and fission yeast, CTU2 knockout causes thermosensitive viability loss, accompanied by significant aberrant development, which could result from both misreading and frameshifting during translation (13). It has been reported to regulate plant immunity through translation reprogramming (18). In Arabidopsis, mutations in the CTU2 homolog lead to loss of tRNA thiolation, reducing translation of Non-expressor of Pathogenesis-Related genes 1 (NPR1), the salicylic acid receptor, and compromising salicylic acid signaling. In the Magnaporthe oryzae model system, the absence of CTU2 results in a reduction in translation elongation at AAA/CAA/GAA codons, without affecting their synonymous codons (21). This leads to a decrease in the levels of key proteins enriched in U34 codons, which are crucial for appressorium development and function.

CTU2 has increasingly been shown to play a role in the progression of various tumors (16, 20, 22–24). For instance, CTU2 levels are elevated in breast tumors and support metastasis. Mechanistically, CTU2 promotes cellular invasion through codon-biased translation of DEK (a DNA-binding oncoprotein), whose coding region is rich in U34 codons, thereby enhancing Internal Ribosome Entry Site (IRES)-dependent translation of the pro-invasive transcription factor Lymphoid enhancer-binding factor 1 (LEF1) (16). Furthermore, studies have found that CTU2 is highly expressed in BRAFV600E-expressing melanoma cells, potentially promoting glycolysis by codon-biased regulation of HIF1α mRNA translation, which is rich in U34 codons, and maintaining high levels of HIF1α protein. This may contribute to melanoma’s acquired resistance to MAPK therapeutic agents (22). Recent research has elucidated the role of CTU2 in hepatocellular carcinoma development and its upstream transcriptional regulatory mechanisms, identifying it as a Liver X receptor (LXR) target gene. Mechanistically, CTU2 enhances lipogenesis by directly promoting the synthesis of lipogenic proteins, providing a novel mechanism for LXR-mediated lipid synthesis regulation (25).

Given the emerging novel role of tRNA in actively regulating gene expression and the crucial role of CTU2-mediated mcm^5^s^2^U tRNA modification, a comprehensive analysis of CTU2 in multiple cancers is extremely necessary.

## 2 Materials and methods

### 2.1 Pan-cancer data collection and processing

Phenotype data of pan-cancer in The Cancer Genome Atlas (TCGA) and normal tissues in Genotype-Tissue Expression (GTEx) database were downloaded from the UCSC Xena Browser (https://xenabrowser.net/). The Gene Expression Omnibus (GEO) database (https://www.ncbi.nlm.nih.gov/geo/) was used to obtain GSE115002 (26), GSE39582 (27), GSE161533 (28), GSE16449 (29), GSE36376 (30), GSE10927 (31), GSE50428 (32), GSE36376 (33), and GSE75037 (34). The proteomics data of multiple cancer types were obtained from the Clinical Proteomic Tumor Analysis Consortium (CPTAC) database (https://proteomics.cancer.gov/programs/cptac). Immunohistochemistry (IHC) images showing CTU2 expression in normal and cancer tissues were retrieved from the Human Protein Atlas (HPA) database (https://www.proteinatlas.org/).

The cBioPortal for Cancer Genomics (http://www.cbioportal.org) was used as a source of merged CTU2 methylation data. UALCAN (http://ualcan.path.uab.edu/analysis.html) was used to explore the promoter DNA methylation levels in CTU2 in normal and pan-cancer tissues. The log_2_ (TPM + 0.001) transformed normalized expression profiles, copy number variations on gene expression were estimated using the GISTIC2.0 method.

### 2.2 Single-cell expression and spatial transcriptomes analysis of CTU2

The single-cell expression levels of CTU2 across various pan-cancer tissues using the Tumor Immune Single-cell Hub (TISCH) database (http://tisch.comp-genomics.org/home/), which also provided UMAP plots illustrating CTU2 expression patterns across different cell types. Spatial transcriptome data were obtained from the 10xGenomics website, BRCA (GSE210616) and PAAD (GSE211895). The Spatial-FeaturePlot function from the Seurat package was used to visualize enrichment scores for each cell type.

### 2.3 Prognosis analysis

The survival information of pan-cancer, including overall survival (OS), progression-free interval (PFI), disease-free interval (DFI) and disease-specific survival (DSS), was downloaded from the TCGA database. The R packages ‘survival’ and ‘survminer’ were used to perform Cox analysis and to generate Kaplan-Meier (KM) survival curves to analyze the association between the expression of CTU2 and patient prognosis.

### 2.4 Immune-related analysis

The ESTIMATE algorithm (https://bioinformatics.mdanderson.org/*estimate*/) was used to compute Immune, Stromal, and ESTIMATE score values for 33 cancer types (35). Utilizing the TIMER2.0 (http://timer.cistrome.org/), we investigated the abundance of various cell types within the tumor microenvironment across 33 cancer types. A total of 11 immune checkpoint genes (including PDCD1, CTLA4, VSIR, HAVCR2, LAG3, TIGIT, SIRPA, BTLA, SIGLEC7, LILRB2, and LILRB4) were extracted from TCGA datasets for correlation analysis of immune checkpoint genes (36). In addition, CTU2 was analyzed in relation to tumor immunity in the following areas, including immune activation, chemokines, chemokine receptors, and major histocompatibility complex (MHC). All gene markers were obtained from previous studies (36–38). The impact of CTU2 expression level on the status of anti-cancer immunity was analyzed in 33 cancer types using the Tracking Tumor Immuno phenotype (TIP) database (http://biocc.hrbmu.edu.cn/TIP). The TIDE website (http://tide.dfci.harvard.edu) was used to retrieve the TIDE score for each patient.

### 2.5 Drug sensitivity analysis

The Genomics of Drug Sensitivity in Cancer (GDSC) database, established by the Sanger Research Institute, gathers data on how tumor cells respond to various drugs (39). The ‘oncoPredict’ tool utilized the GDSC V2 database to assess the drug sensitivity of samples in both the training and validation datasets (40). The CMAP_gene_signatures. RData file, which contains 1288 compounds-related signatures, was downloaded from https://www.pmgenomics.ca/bhklab/sites/default/files/downloads, and used for calculating the matching score. We constructed a gene-related signature consisting of the 150 most significantly upregulated and the 150 most significantly downregulated genes, determined by comparing patients with high and low gene expression in tumors. Using the optimal feature matching method XSum (eXtreme Sum), we compared the gene-related features with cMAP gene features to obtain similarity scores for 1,288 compounds. The analysis process was followed the methodology outlined in previous publications (41, 42).

### 2.6 Gene Set Enrichment Analysis (GSEA) and correlation analysis

To evaluate the biological function of a single gene in tumors, Pearson’s correlation analysis was performed to examine the relationship between CTU2 expression and other mRNAs using TCGA transcriptome data. Genes with the highest correlation with CTU2 expression were selected for enrichment analysis. GSEA was conducted using the R package ‘clusterProfiler’, based on predefined gene sets from the Molecular Signatures Database v5.0 (http://software.broadinstitute.org/gsea/msigdb/index.jsp). For this study, the ‘c2.cp.kegg.v7.5.1.entrez.gmt’ and ‘c5.go.bp.v7.5.1.entrez.gmt’ collection sets were utilized in the GSEA.

### 2.7 DepMap (The Cancer Dependency Map) analysis

For a diverse set of pan-cancer cell lines, gene-level essentiality scores (obtained from CRISPR knockout and RNAi knockdown screens) were extracted from the from the DepMap Public 21Q3 dataset using the DepMap portal (depmap.org/portal). For REACTOME gene sets (acquired from MSigDB v7.4), Student’s t-tests were performed to compare the false discovery rate (FDR) values of genes within each gene set to those outside it. The gene set dependency score was computed by multiplying the FDR value for each gene set by the sign of its corresponding t-statistic.

### 2.8 Cell culture

Given the expression and prognostic significance of CTU2 across various cancer types, particularly considering the high incidence and mortality of kidney renal clear cell carcinoma (KIRC) and liver hepatocellular carcinoma (LIHC), representative cell lines from these malignancies were selected for functional validation. The human liver cancer cell line Huh-7, human renal clear cell carcinoma cell line 786-O and murine liver cancer cell line Hepa1–6 were obtained from the American Type Culture Collection. Both cell lines were cultured in complete DMEM medium (Thermo Scientific, Waltham), supplemented with 10% fetal bovine serum (FBS, Gibco, Thermo Scientific, Waltham), at 37°C in a 5% CO2 incubator.

### 2.9 Stable cell line construction

The shRNA sequences targeting human CTU2 gene, following the sequences shRNA-1: GTTCCTTCTGTCTTCACACCA; and shRNA-2: GAAGTGTGTGAAGTGCAAGGA, were obtained from Genechem (Shanghai, China) and were constructed into lentiviruses backbone plasmid. The shRNA sequences targeting mouse CTU2 gene are described in refs (22). A scrambled non-specific control shRNA sequence was also cloned into the same vector and used as a control. Huh-7, 786-O and Hepa1–6 cell lines were planted in six-well plates 24 h before transfection at the cell density of 2 × 10^5^ cells/well. Lentivirus packaging was carried out following previously established protocols (43). Stable cell lines were generated by infecting cell cultures with lentivirus.

### 2.10 Colony formation assay

After the stable CTU2 knockdown cell lines were successfully constructed, the cells were seeded in six-well plates at densities of 1500 cells/well, and the cells were cultured for 2 weeks. Finally, the cells were fixed with 4% para-formaldehyde and stained with crystal violet, and colonies containing more than 50 cells were counted and analyzed.

### 2.11 Western blotting

The cells were lysed with ice-cold RIPA lysis buffer (Servicebio, China) containing protease inhibitors and centrifuged at 4°C (12,000 rpm, 20 min). The protein supernatant was then quantified using a BCA protein assay kit (Biyuntian, China). Following protein denaturation, 30 μg of protein was separated by SDS-PAGE on 10% gels and transferred to a PVDF membrane (Millipore, USA). After blocking with 5% skim milk in TBS-T, the membrane was incubated overnight at 4°C with the following antibodies: anti-CTU2 (ab177160, 1:1000), anti-USF1 (ab125020, 1:1000), and anti-GAPDH (Proteintech, 60004-1-Ig). The membrane was then incubated with goat anti-rabbit (Proteintech, RGAR001, 1:5000) or mouse IgG secondary antibodies (Proteintech, RGAM001, 1:5000) for 1 hour. Following this, the membranes were washed three times with TBS-T (5 min per wash) and visualized using an enhanced chemiluminescence substrate.

### 2.12 EdU proliferation assay

EdU detection was performed using the EdU Imaging Kits (APEXBIO, K1076, USA) according to the manufacturer’s protocol. Briefly, cells were incubated with 10 μM EdU for 1 hour, then trypsinized, washed with PBS, fixed with 4% paraformaldehyde (PFA) for 20 minutes, and permeabilized with 0.1% Triton X-100 for 20 minutes. The single-cell suspensions were washed twice with PBS and incubated with the appropriate EdU flow cytometry antibodies for 30 minutes in the dark at room temperature. The EdU-positive rate was calculated as follows: EdU-positive rate = (EdU-positive cell count/(EdU-positive cell count + EdU-negative cell count)) × 100%.

### 2.13 Flow cytometric analysis of cell apoptosis

For apoptosis assays, the Alexa Fluor 488 Annexin V/PI Cell Apoptosis Kit (Vazyme, A211-01, China) was used according to the manufacturer’s instructions. The established stable cell lines were digested with EDTA-free trypsin, washed with PBS, and stained with Annexin V-Alexa Fluor 488 (FITC) and propidium iodide (PI) as recommended. Flow cytometry was then performed according to the manufacturer’s guidelines, and the proportion of apoptotic cells (early apoptosis plus late apoptosis) was calculated.

### 2.14 Luciferase reporter assay

CTU2 wild-type and mutant dual-luciferase reporter gene plasmids were constructed based on the base sequence by You Bao Biotechnology (Changsha, China). The dual-luciferase reporter assay was conducted using the Dual-Luciferase Reporter Assay System (Vazyme, DD1205, China). Cells were plated in 12-well plates at a density of 2 × 10^5^ cells per well and transfected with Lipofectamine 3000. After 24 hours of transfection, Firefly and Renilla luciferase activities were measured according to the manufacturer’s instructions. Firefly luciferase activities were normalized to Renilla luciferase activities, and the ratio of Firefly to Renilla luminescence was calculated.

### 2.15 Migration and invasion assays

Cell migration and invasion assays were conducted using Transwell chambers (8-μm pore size, Corning, USA). The lower compartment of the Transwell chamber was filled with 600 μl DMEM containing 10% FBS, and a 100 μl serum-free cell suspension containing 8×10^4^ cells was seeded into the upper chamber. For the invasion assay, matrigel-coated invasion chambers were utilized to evaluate cell invasion.

### 2.16 *In vivo* LIHC murine models

All animal experiments in this study were performed in accordance with the guidelines for the welfare and ethics of experimental animals of Zhejiang University with the approval of the Animal Experimental Ethics Committee of Zhejiang University. Female nude mice (BALB/c, 6 weeks old) were obtained from GemPharmatech (Jiangsu, China) and housed in a specific-pathogen-free (SPF) animal facility. For the subcutaneous tumor xenograft models, mice were randomly assigned to three groups (6 mice per group): shNC, shCTU2-1, and shCTU2-2. Each nude mouse received a subcutaneous inoculation of 1 × 10^7 cells (100 μL) in the right hind limb. Tumor size was measured using Vernier calipers every five days, and tumor volume was calculated as V = (Length × Width^2)/2. Mice were euthanized when the maximum tumor volume reached 1500 mm^3, and tumors were harvested, weighed, and imaged.

An orthotopic LIHC tumor model was established by implanting 5×10^6^ Hepa1–6 cells directly into the liver of C57BL/6 male mice (6–8 weeks old, GemPharmatech). Three weeks after inoculation, the mice were euthanized, and the tumor nodules in the liver were quantified and measured.

### 2.17 Flow cytometry analysis of orthotopic LIHC tumor nodules

Single-cell suspensions were generated from orthotopic liver of tumor-bearing mice. The following anti-mouse antibodies were used: FITC-Anti-CD11b (cat# 101205), BV605-Anti-Gr-1 (cat# 563299), APC-Cy7-Anti-MHC-II (cat# 107629), BV421-Anti-CD11c (cat# 117329), Percp-Cy5.5-Anti-CD8 (cat# 100733) and APC-Anti-PD-1 (cat# 100733) was purchased from Biolegend (San Diego, CA). Cells were analyzed using with CyAnADP analyzer (Beckman Coulter).

### 2.18 APM-dPAGE and Northern blot

To isolate single tRNA Lys, small RNAs (≤200 nt) were extracted using the MiPure cell miRNA Kit (Vazyme, RC201, China). The presence of the mcm^5^s^2^U modification in tRNAs was confirmed by observing reduced electrophoretic mobility in a 10% polyacrylamide gel containing 0.05 mg/ml [(N acryloyl amino) phenyl] mercuric chloride (APM) and 7 M urea, were performed as described (44). Subsequently, the APM-PAGE gels were transferred onto positively charged Nylon membranes (Roche, USA). Membranes containing tRNA were hybridized with DIG-labeled probes synthesized by Sangon Biotech (Shanghai, China), following the sequences: TAAAAGTCTGATGCTCTACC. The RNA from hydrogen peroxide (H2O2) pre-treatment served as a negative control for desulfurization.

### 2.19 Electrophoretic Mobility Shift Assay (EMSA)

Nuclear extracts from huh-7 cells were prepared using the Nuclear Extract Kit (Active Motif, CA) according to the manufacturer’s instructions. The DNA-binding activity of USF1 in the nuclear extracts was assessed using the Light-Shift EMSA Optimization and Control Kit (Thermo Scientific, USA). A biotin-labeled wild-type oligonucleotide probe corresponding to the USF1 E-box motif was designed as follows: 5’- GGGCGGGCGCGCTCACGTGTGGCCGCAGCTG-3’. Additionally, an unlabeled wild-type probe (without biotin) was designed and used in the competition reaction. A mutated E-box motif probe, also unlabeled, was constructed with the following sequence: 5’-GGGCGGGCGCGCTAAAAAAAGGCCGCAGCTG-3’. The DNA-protein complexes were resolved by electrophoresis on a 6% polyacrylamide gel, followed by visualization and analysis of band shifts via autoradiography.

### 2.20 Statistical analysis

Correlation analysis was performed using Spearman’s rank correlation. The *in vitro* experiments were conducted in triplicate. All statistical analyses were carried out using GraphPad Prism 7.0, SPSS (version 22.0), or R software (version 4.1.2). *P value* of < 0.05 was considered statistically significant. Statistical significance is indicated as follows: ns (not significant), *P < 0.05, **P < 0.01 and ***P<0.001.

## 3 Results

### 3.1 CTU2 is upregulated across multiple cancer types

**Figure 1:**
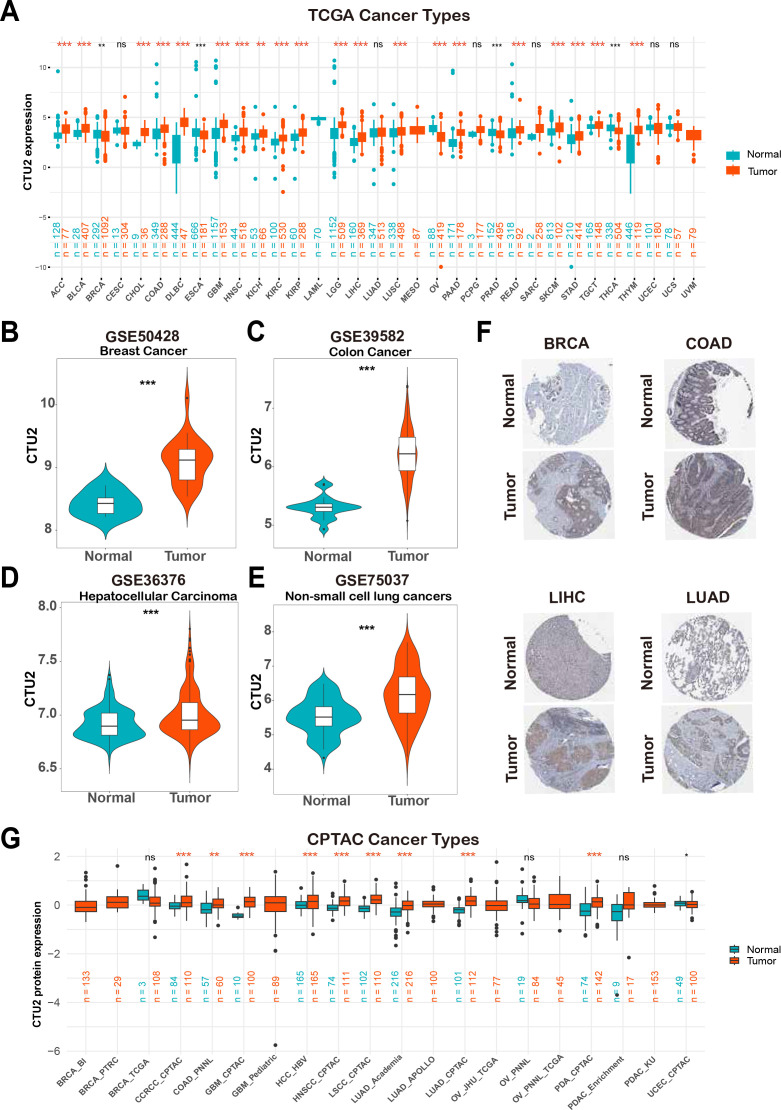
Upregulation of CTU2 across multiple cancer types. **(A)** Analysis of CTU2 mRNA expression across 33 cancer types using the TCGA and GTEx databases; **(B–E)** Differential CTU2 mRNA expression in various cancer GEO datasets; **(F)** Representative images of CTU2 protein expression in normal and tumor tissues of the breast, colon, liver, and lung from the HPA database; **(G)** CTU2 protein expression analysis in 12 cancer types using data from the CPTAC database. The red asterisk (*) indicates a significant upregulation. *P < 0.05, **P < 0.01, ***P < 0.001, and ns (not significant).

Initially, the TCGA and GTEx databases were utilized for a comprehensive pan-cancer analysis of CTU2 mRNA expression profiles. This investigation revealed significant differential expression of CTU2 across 24 cancer types (**Figure 1A**), with fold changes exceeding 2 in diffuse large B-cell lymphoma (DLBC), thymoma (THYM), cholangiocarcinoma (CHOL), and glioblastoma multiforme (GBM) (**Supplementary Table S1**). Paired Student’s t-test further demonstrated a significant increase in CTU2 expression in multiple tumor tissues compared to adjacent normal tissues (**Supplementary Figures S1A–1M**). Analysis of seven GEO datasets confirmed elevated CTU2 expression in breast cancer (BRCA), colon adenocarcinoma (COAD), LIHC, non-small cell lung cancer (NSCLC) (**Figures 1B–E**), esophageal cancer (ESCA), KIRC, and adrenocortical carcinoma (ACC) (**Supplementary Figures S1N–P**). Consistently, immunohistochemical data from the HPA databases confirmed increased CTU2 protein levels in BRCA, COAD, LIHC, and lung adenocarcinoma (LUAD) (**Figure 1F**). At the protein level, CTU2 was upregulated in 9 datasets across 8 cancer types in the CPTAC database, including clear cell renal cell carcinoma (CCRCC), COAD, GBM, hepatocellular carcinoma (HCC), head and neck squamous cell carcinoma (HNSC), lung squamous cell carcinoma (LSCC), LUAD, and pancreatic ductal adenocarcinoma (PDA) (**Figure 1G**).

### 3.2 Overall landscapes of single-cell expression levels and spatial transcriptomics of CTU2

**Figure 2:**
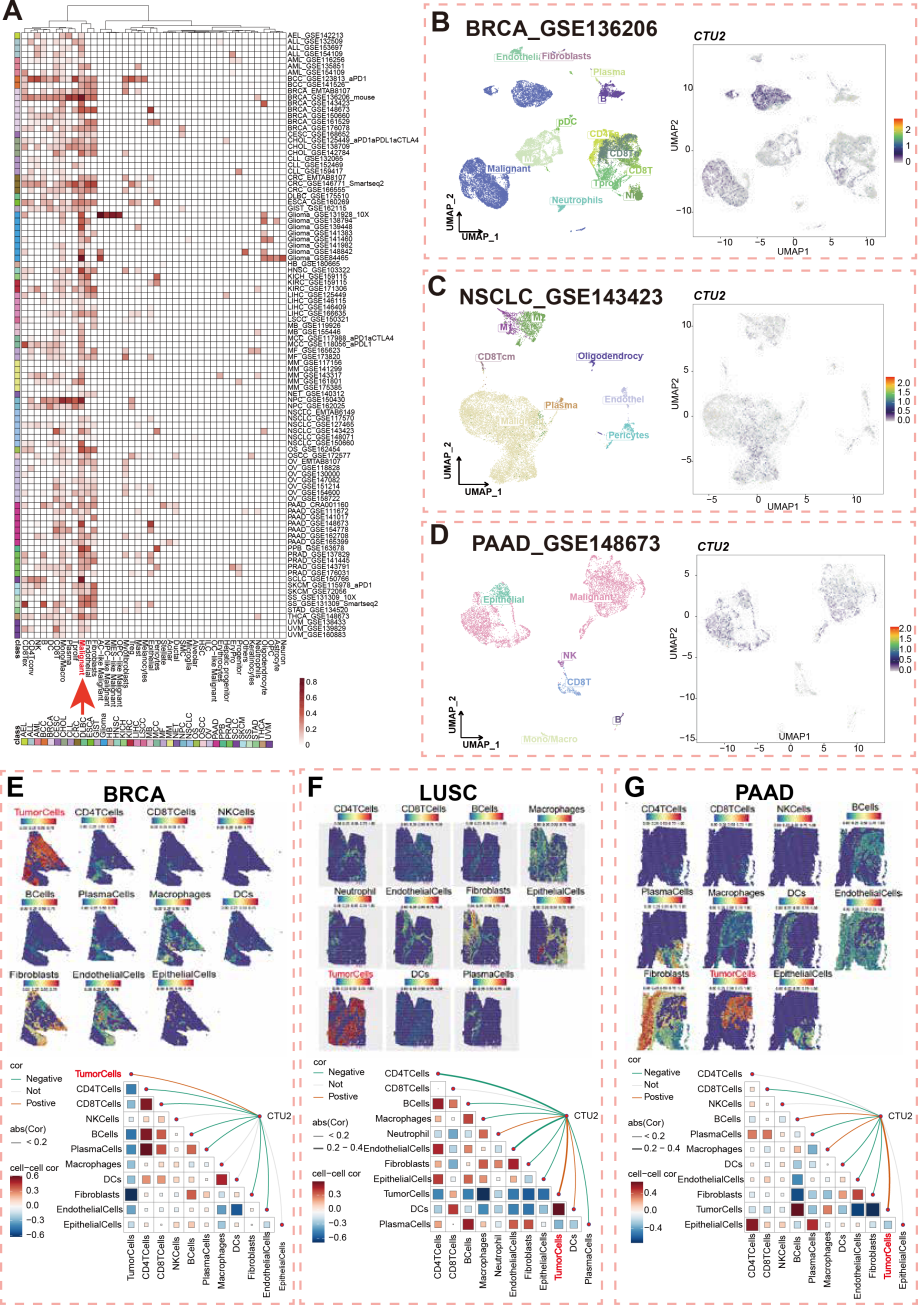
Single-cell and Spatial transcriptomics of CTU2 expression across multiple cancer types. **(A)** Cluster heatmaps showing the mRNA expression pattern of CTU2 in different cell types across different tumor types; **(B)** Umap plots displaying the clustering of different cell types (left panel) and CTU2 expression level (right panel) in BRCA **(B)**, NSCLC **(C)**, and PAAD tissues; Upper Spatial transcriptomics deconvolution maps visualize cell localization in BRCA **(E)**, LUSC **(F)** and PAAD **(G)**. Color ranging from blue to red represents the abundance of that cell type within the spot. Lower correlation analysis calculates the relationships between cell abundances and CTU2 expression levels. Red lines indicate positive correlations, green lines denote negative correlations, and gray lines represent non-significant correlations. The thickness of the lines reflects the absolute value of the correlation coefficients. The correlation in triangular regions is represented by the color intensity and size of the squares: red indicates a positive correlation, blue indicates a negative correlation and darker colors signify more significant *p-values*. Larger squares correspond to greater absolute values of the correlation coefficients.

We analyzed the TISCH database to illustrate the landscape of CTU2 single-cell expression. Among 98 single-cell sequencing datasets, we found that CTU2 expression is predominantly observed in the malignant cell types of most tumors (**Figure 2A**, red arrow). We randomly selected common tumor types for specific analysis, and the UMAP plots of BRCA, NSCLC, and pancreatic adenocarcinoma (PAAD) datasets intuitively showed that CTU2 is mainly expressed in malignant cells (**Figures 2B–D**). Specifically, in BRCA (GSE136206), UMAP plots (**Figure 2B**, left panel) revealed CTU2 expression in various cell types, including malignant cells, endothelial cells, fibroblasts, monocytes, macrophages, CD4^+^ T cells, CD8^+^ T cells, natural killer (NK) cells, and T-proliferating cells, with particularly high expression levels observed in malignant cells (**Figure 2B**, right panel).

Unlike single-cell sequencing, spatial transcriptomics preserves spatial information while providing insights into gene expression, cell types, and tissue context. Next, we utilized spatial transcriptome data to further assess the spatial distribution of CTU2 and malignant cells in BRCA, lung squamous cell carcinoma (LUSC), and PAAD. Spatial infiltration heatmaps revealed that different sequencing spots were annotated with distinct cell types, including malignant cells, fibroblasts, and key immune cells (CD4^+^ T cells, CD8^+^ T cells, NK cells, B cells, and dendritic cells) (**Figures 2E–G**, upper panel). Spearman correlation analysis demonstrated a significant positive correlation between CTU2 expression and tumor cell density in specific regions, indicating that CTU2^+^ cells were primarily clustered in regions populated by malignant cells (**Figures 2E–G**, lower panel). In the spatial transcriptomics data of LIHC and skin cutaneous melanoma (SKCM), CTU2 is also primarily expressed in tumor tissue regions (**Supplementary Figure S2**). These results emphasize that CTU2 is mainly expressed by tumor cells in pan-cancer and its potential as a therapeutic target.

### 3.3 Prognostic role of CTU2 in human cancers

**Figure 3:**
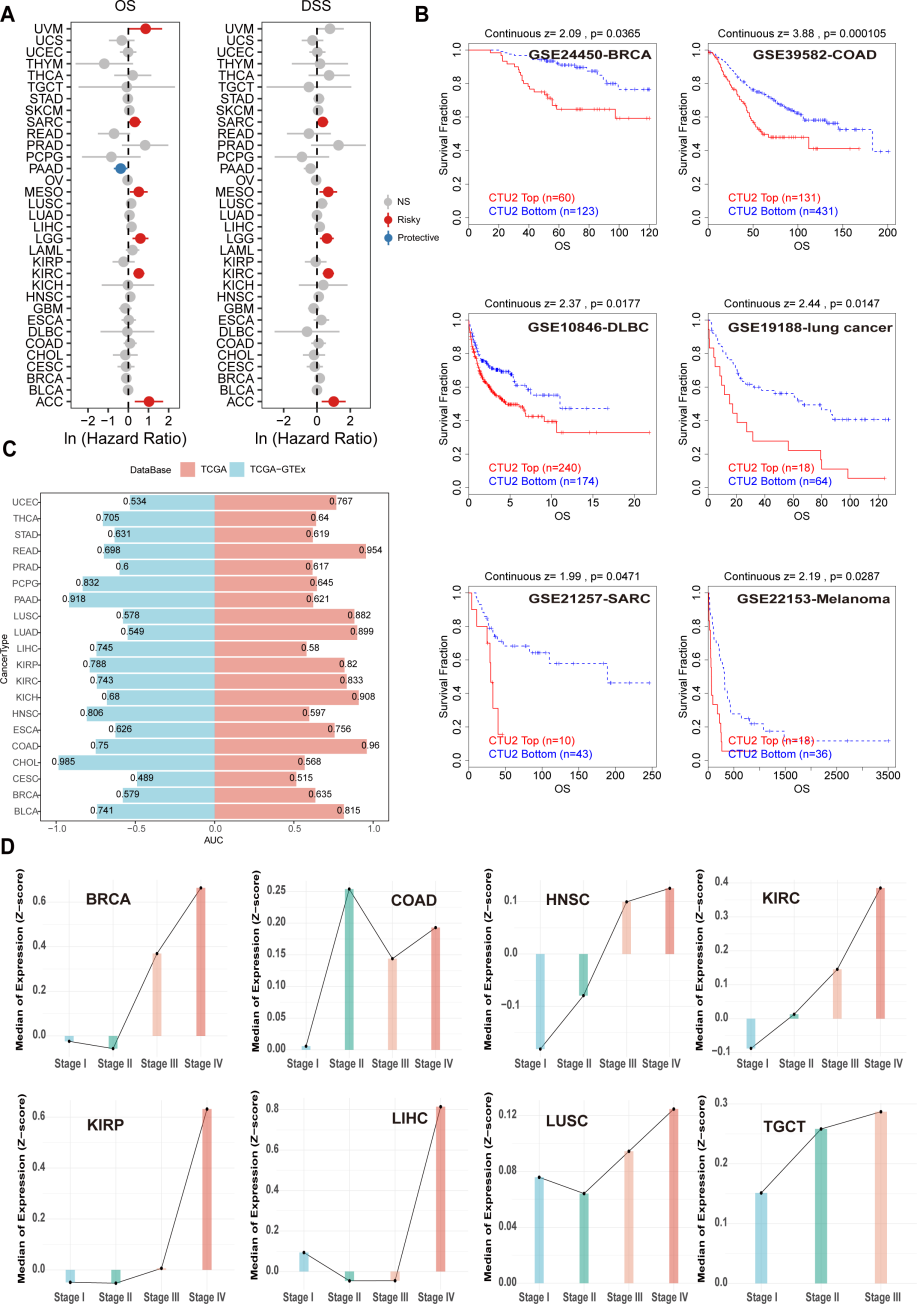
Correlation between CTU2 expression and pan-cancer prognosis and diagnosis. **(A)** OS and DSS associated with CTU2 expression in 33 cancer types from TCGA; **(B)** Kaplan-Meier analysis of OS based on CTU2 mRNA expression across multiple tumors using the TIDE tool; **(C)** AUC values from receiver operating ROC analysis; blue indicates the TCGA-GTEx cohort, while red represents the TCGA cohort; **(D)** CTU2 mRNA expression across different tumor stages in various cancers.

Univariate Cox regression analyses revealed that high CTU2 mRNA expression was significantly associated with OS and DSS across multiple cancers, particularly in ACC, KIRC, lower-grade glioma (LGG), mesothelioma (MESO), and sarcoma (SARC) (**Figure 3A**). These associations were further supported by DFI and PFI analyses, primarily in LIHC and SARC (**Supplementary Figure S3A**). Kaplan-Meier curves indicated that elevated CTU2 mRNA expression correlated with poor prognosis in ACC, KIRC, LGG, LIHC, SARC, uveal melanoma (UVM), thyroid cancer (THCA) and LUSC (**Supplementary Figures S3B, C**). Similarly, CPTAC data indicated that high CTU2 protein levels correlated with poor prognosis in BRCA, LIHC, LUAD, and KIRC (**Supplementary Figure S3D**). Multiple GEO datasets from the TIDE website further validated poor prognosis in patients with high CTU2 mRNA levels in BRCA, COAD, DLBC, LUAD, SARC, and melanoma (**Figure 3B**). ROC curve analysis demonstrated that CTU2 has high diagnostic accuracy (AUC > 0.8) for eight cancer types, including READ, LUSC, LUAD, kidney renal papillary cell carcinoma (KIRP), KIRC, kidney chromophobe (KICH), COAD, and bladder urothelial carcinoma (BLCA) (**Figure 3C**). Integrating TCGA and GTEx data further supported CTU2’s diagnostic potential in pheochromocytoma and paraganglioma (PCPG), PAAD, HNSC, and CHOL (**Figure 3C**).

We also analyzed clinical phenotype data from TCGA to investigate CTU2 mRNA expression patterns across different clinical stages and their association with clinical features in various cancers. CTU2 mRNA levels increased with advancing clinical stage in cancers such as BRCA, HNSC, KIRC, KIRP, LIHC, LUSC, and testicular germ cell tumors (TGCT) (**Figure 3D**). The CPTAC database indicates that in BRCA, CCRCC, LSCC, and LUAD, CTU2 protein levels are elevated in Stage IV (late-stage) compared to earlier stages (Stage I) (**Supplementary Figure S3E**). We further examined CTU2 expression across different molecular tumor subtypes and found distinct gene expression profiles for specific cancers (**Supplementary Figure S3F**). Additionally, the expression of CTU2 was found to be correlated with T stage, N stage, and M stage in various cancers (**Supplementary Figures S4A–H**). These findings suggest that CTU2 could be a significant and potential tumor marker across multiple cancers.

### 3.4 Alterations of CTU2 modified tRNA expression across cancer types

Given the role of CTU2 across cancers, we next map the expression profile of its modified tRNAs in a pan-cancer context. High-throughput quantification of tRNAs is challenging due to extensive post-transcriptional modifications and complex secondary structures. To overcome this, as reported in the literature, we utilized microRNA-sequencing data from the TCGA database, which includes data from approximately 10,000 patients, as an alternative method for quantifying tRNA expression (**Supplementary Table S2**). The mcm^5^s^2^U modification, mediated by CTU2 at the wobble position, restricts and constrains the strict complementary pairing between the anticodon (tRNA-Glu-TTC, tRNA-Lys-TTT, tRNA-Gln-TTG) and its corresponding codon (GAA, AAA, CAA), despite the wobble pairing expands the decoding ability of tRNAs (8).

**Figure 4:**
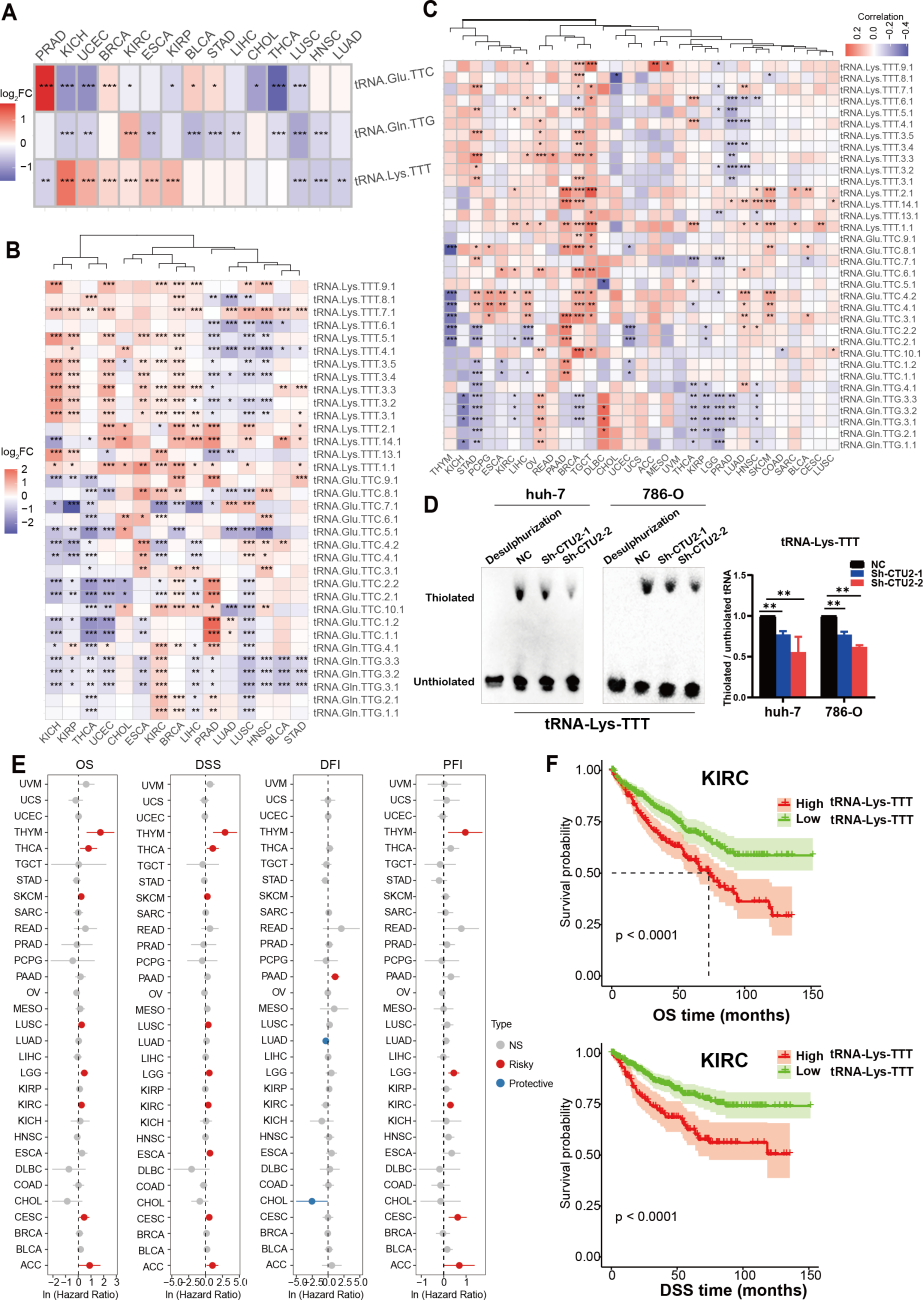
Expression characteristics of CTU2 specific-modified tRNAs in different cancer types. **(A)** Expression characteristics of the three specific-modified tRNAs in different cancer types, with colors ranging from blue to red representing the log_2_FC values; **(B)** Expression characteristics of the tRNA isoforms in different cancer types, with colors ranging from blue to red representing the log_2_FC values; **(C)** Heatmap showcases the specific-modified tRNAs correlated with CTU2 based on correlation analysis; **(D)** Northern blot analysis was performed to assess the mcm^5^s²U modification of tRNA-Lys-TTT in CTU2 knockdown and control huh-7 and 786-O cells (slow-migration band indicates thiolated tRNA). No retarded band was observed after desulphurization. The mcm^5^s²U modification level was normalized as the ratio of thiolated to unthiolated tRNA. The graph on the right represents the statistical analysis of gray values. The experiment was repeated independently three times; **(E)** OS, DSS, DFI and PFI of tRNA-Lys-TTT in 33 TCGA cancer types; **(F)** Kaplan-Meier analysis of OS and DSS for tRNA-Lys-TTT expression in KIRC. *P < 0.05, **P < 0.01, and ***P < 0.001.

We first examined differential expression of the three modified tRNAs and their isoforms between paired tumor and normal samples, finding that tRNA-Lys-TTT (**Figure 4A**) and its isoforms (**Figure 4B**) were highly expressed in multiple cancer types, notably in KICH, uterine corpus endometrial carcinoma (UCEC), BRCA, KIRC, ESCA, and KIRP. Further correlation analysis revealed a significant positive association between CTU2 expression and the expression of multiple isoforms of tRNA-Lys-TTT across various tumors, especially in BRCA, LIHC, stomach cancer (STAD), OV and TGCT (**Figure 4C**). The tRNA mcm^5^s^2^U modification, a form of thiouridine modification, was evaluated by electrophoretic mobility retardation using Northern blot (45, 46). *In vitro* results confirmed that CTU2 knockdown reduced mcm^5^s²U modification levels on tRNA-Lys-TTT in LIHC (huh-7) cells and KIRC (786-O), as indicated by decreased thiolation of the target tRNA (**Figure 4D**). We also found that tRNA-Lys-TTT expression was linked to OS and DSS in multiple tumors (**Figure 4E**). These findings suggest that tRNA-Lys-TTT expression could serve as a prognostic marker, with KIRC as an example (**Figure 4F**). Thus, not only does CTU2 contribute to cancer progression, but its modified tRNA is also linked to poor prognosis in various tumors.

### 3.5 Impact of CTU2 expression on the tumor microenvironment in pan-cancer

**Figure 5:**
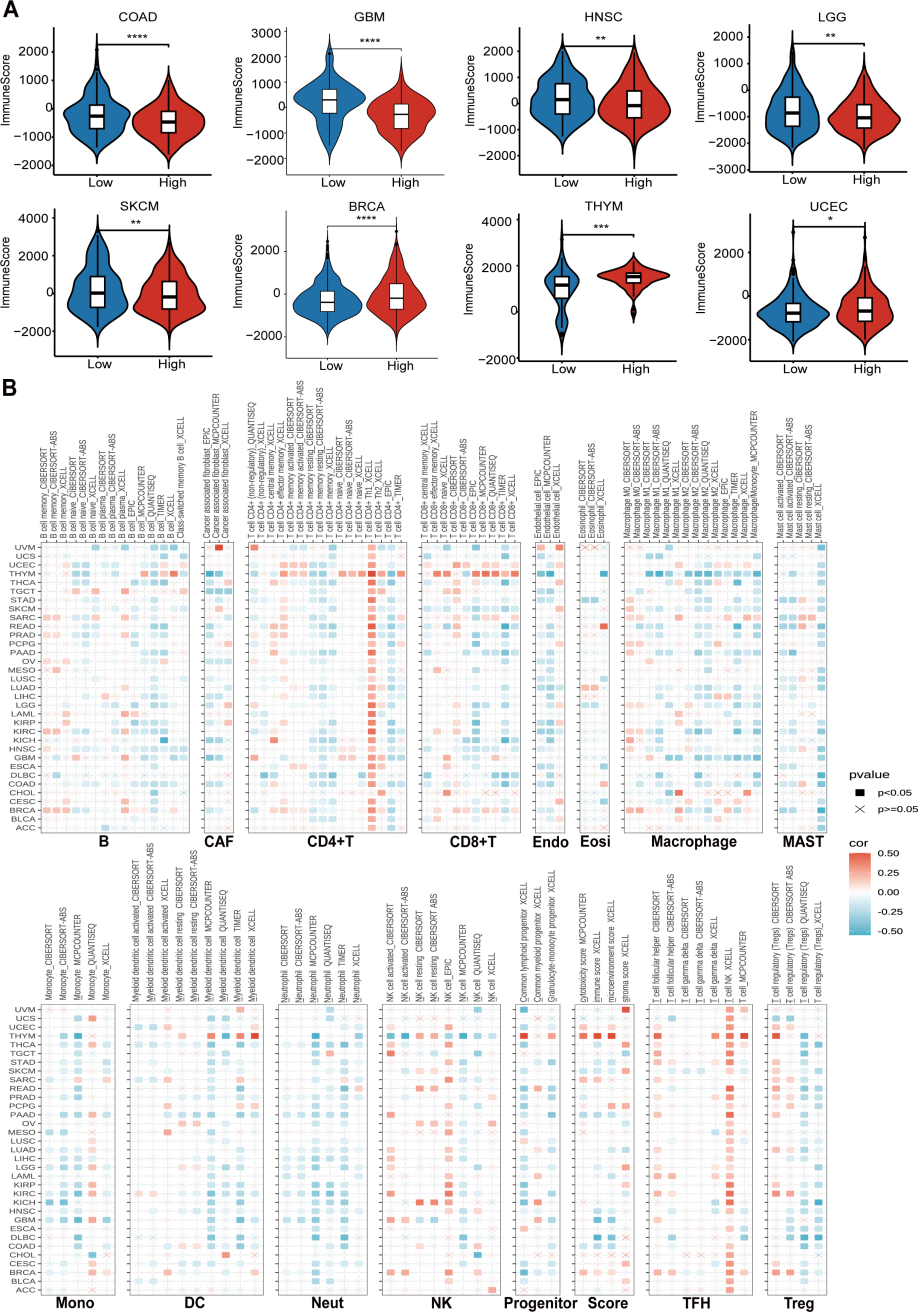
CTU2 contributes to diverse immune cell infiltration in various types of cancer. **(A)** Boxplots show the comparison of immune scores between CTU2-high and CTU2-low patients, distinguished by the median; **(B)** Cluster heatmaps display the correlation between CTU2 expressions and the degree of infiltration by B, cancer-associated fibroblast (CAF), CD4^+^T, CD8^+^T, endothelial (Endo), eosinophil (Eosi), macrophage, MAST, monocyte (Mono), DC, neutrophil (Neut), NK, progenitors, TFH, and Treg. *P < 0.05, **P < 0.01, and ***P < 0.001.

Firstly, we utilized the ESTIMATE database to investigate the impact of CTU2 expression on immune cell infiltration in human cancers (**Supplementary Table S3**). It is worth noting that in most tumors, including COAD, GBM, HNSC, LGG, and SKCM, high CTU2 expression was associated with lower immune scores, suggesting that elevated CTU2 expression in these tumors may indicate reduced immune infiltration. Conversely, in BRCA and UCEC, high CTU2 expression was correlated with higher immune scores, implying greater immune infiltration (**Supplementary Figure S5A**, **Figure 5A**). We also utilized the TIMER 2.0 database to explore the correlation between CTU2 expression and the infiltration of specific immune cell types across various cancers. Our analysis revealed that, in most tumor types, tumor CTU2 expression is negatively correlated with the infiltration of major immune cell subtypes, such as CD8^+^ T cells and DC cells (**Figure 5B**). These findings suggested that CTU2 expression in tumor cells may play a role in modulating the migration and infiltration of immune cells, potentially influencing the response to immunotherapy in human cancers.

### 3.6 Predictive potential of CTU2 in cancer immunotherapy response

**Figure 6:**
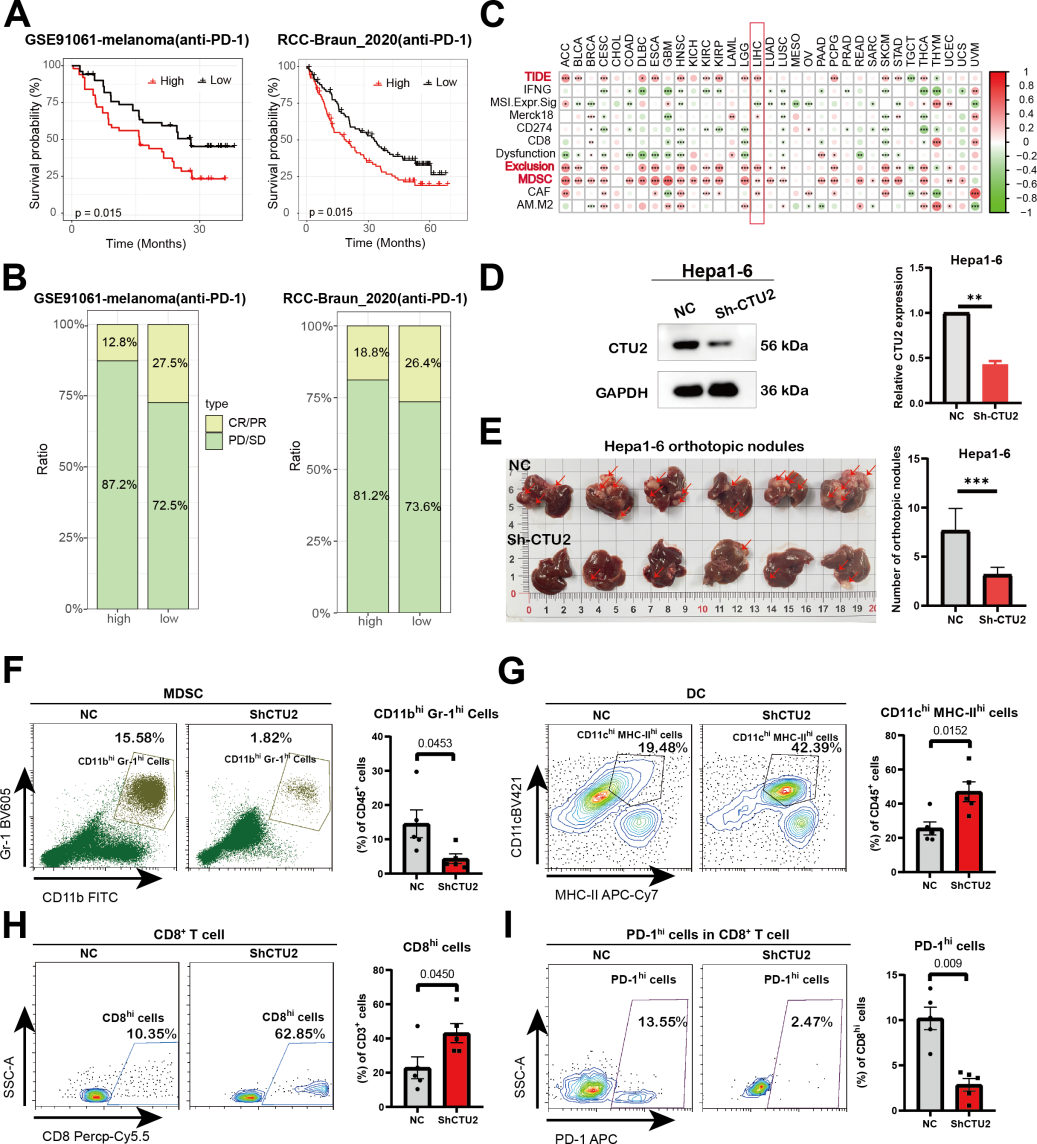
Influence of CTU2 expression on anti-tumor immunity and immunotherapy response. **(A)** Predictive values of CTU2 expression on OS of melanoma (left) and renal cell carcinoma (right) patients in anti-PD-1 immunotherapy; **(B)** Response rate of immunotherapy in melanoma (left) and renal cell carcinoma (right) patients, PD means progressive disease, SD means stable disease, CR means complete response, and PR refers to partial response; **(C)** The correlation heatmap shows the correlation between CTU2 expression and TIDE scores with the TIDE tool; **(D)** The results of western blotting confirmed the knockdown effect of CTU2 in Hepa1-6. The grey value of the CTU2 protein levels was normalized to that of the corresponding GAPDH (right panel). The experiment was independently repeated three times (**P value< 0.01); **(E)** Representative pictures of Hepa1–6 liver orthotopic tumor lesions. Quantification of Hepa1–6 liver orthotopic tumor lesions (n = 6, ***P value< 0.001) was listed in the right panel; **(F-I)** Left: Representative flow cytometry plots of MDSC cells, CD8^+^ T cells, DC cells, and exhausted CD8^+^ T cells (PD-1 high). Right: Statistical quantification of cell numbers (n = 5, *P* value<0.05 were considered statistically significant).

Given the prognostic significance of CTU2 in immune infiltration, we proceeded to investigate its predictive impact on cancer immunotherapy response. We first investigated the predictive value of CTU2 in real-world immunotherapy response by incorporating data from two independent immunotherapy studies (GSE91061-melanoma; RCC-Braun_2020) (**Figures 6A, B**). We found that melanoma and kidney cancer patients with high CTU2 expression had poorer survival prognosis and lower response rates to anti-PD-1 immunotherapy (**Figures 6A, B**). However, patients with low CTU2 levels demonstrated a higher likelihood of responding to immunotherapy, as evidenced by improved prognosis in melanoma and renal cell carcinoma when treated with anti-PD-1 therapy, compared to those with high CTU2 levels (**Figures 6A, B**).

Higher TIDE prediction scores indicate a greater likelihood of immune evasion, suggesting that patients are less likely to benefit from immune checkpoint inhibition therapy (ICI therapy) (47, 48). In the TCGA dataset, high CTU2 expression was associated with higher TIDE scores, particularly in ACC, BLCA, cervical squamous cell carcinoma and endocervical adenocarcinoma (CESC), HNSC, ESCA, KIRC, LIHC, LGG, PCPG, SKCM, STAD, THCA, UCEC, and KIRP (**Figure 6C**, **Supplementary Figure S6**). Subsequently, we analyzed the comprehensive mechanism of tumor immune dysfunction and exclusion using the TIDE database. Our findings revealed that high CTU2 expression was associated with increased infiltration of myeloid-derived suppressor cells (MDSCs) and elevated T-cell exclusion scores across multiple cancers, including ACC, BLCA, CESC, DLBC, ESCA, HNSC, KIRC, KIRP, LGG, LIHC, LUAD, LUSC, PCPG, STAD, THCA, and UCEC (**Figure 6C**). The above results suggest that in most tumors, high CTU2 expression may be associated with an immunosuppressive microenvironment.

**Figure 7:**
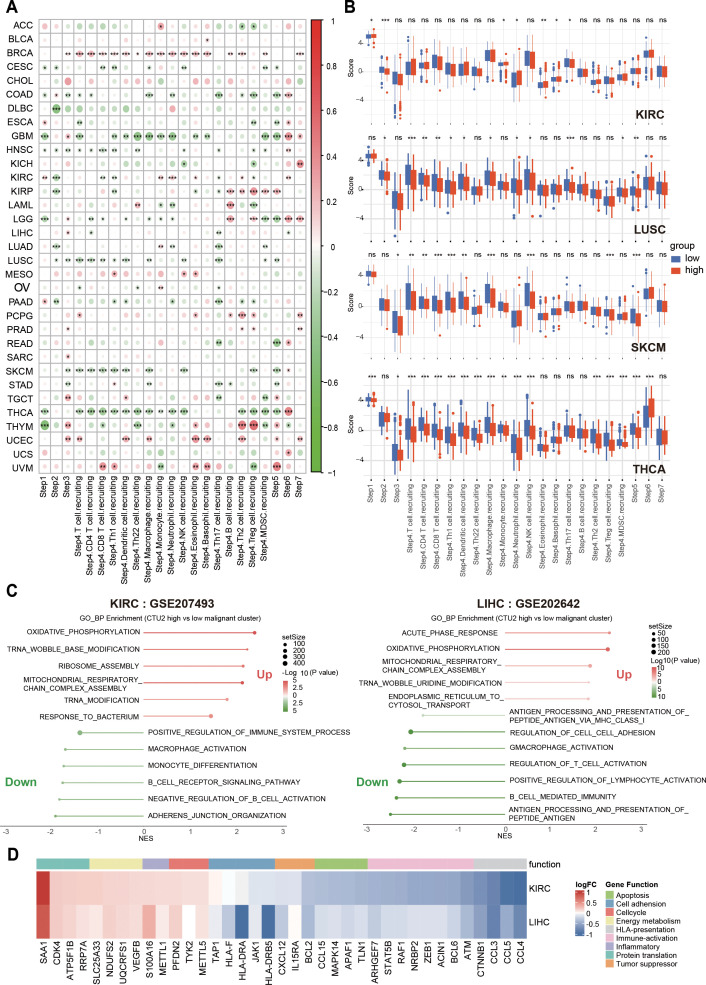
Correlation between CTU2 expression, cancer-immunity cycles, immune suppression, and cancer-related biological processes. **(A)** The correlation heatmap shows the correlation between CTU2 expression and the activity scores of the cancer-immunity cycles; **(B)** Boxplots show the differences in activity scores of the cancer-immunity cycles between CTU2 high-expressing and CTU2 low-expressing tumors in KIRC, LUSC, SKCM, and THCA. **(C)** GSEA pathway enrichment analysis of differentially expressed genes between high CTU2 expression and low CTU2 expression malignant tumor cells; **(D)** Heatmap showing differential expression of key genes involved in important biological function pathways between malignant tumor cells with high CTU2 expression and those with low CTU2 expression. *P < 0.05, **P < 0.01, ***P < 0.001, ****P < 0.0001 and ns (not significant).

To validate this, we conducted *in vivo* experiments and found that knocking down CTU2 expression in Hepa1-6 (mouse liver cancer cell line) significantly reduced the number of tumor lesions in liver cancer orthotopic models (**Figures 6D, E**). Moreover, flow cytometric analysis of liver tumor lesions from the two groups showed that, compared to the NC group, the CTU2 knockdown group exhibited a more active immune microenvironment. This was evidenced by a significant reduction in MDSC numbers, an increase in CD8^+^ T cells and DC cells, along with a decrease in the number of exhausted CD8^+^ T cells (PD-1 high) (**Figures 6F–I**). Activity scores of the cancer-immunity cycles from the TIP database were downloaded and assessed (**Supplementary Table S4**). In addition, as shown in **Figures 7A, B**, the expression of CTU2 affects the tumor immune cycle response differently across various cancers. Additionally, CTU2 expression shows differential correlations with the expression of several key immune checkpoints (**Supplementary Figure S5B**) and various immunomodulators in different tumors (**Supplementary Figure S7**).

To further explore the relationship between tumor CTU2 expression and immune microenvironment infiltration, we analyzed the KIRC single-cell dataset (GSE207493) and the LIHC single-cell dataset (GSE202642). Based on the mRNA expression levels of CTU2 in malignant tumor cells, we classified the tumor cells into two groups: those with high CTU2 expression and those with low CTU2 expression. GSEA was then performed on the differentially expressed genes. Notably, we observed strikingly similar pathway enrichment patterns across these different cancer types (**Figure 7C**). Tumor cells with high CTU2 expression, compared to those with low CTU2 expression, exhibited negative enrichment in immune response-related pathways, including Regulation of T Cell Activation, Antigen Processing and Presentation of Peptide Antigen via MHC Class I, Macrophage Activation, and B Cell Immune Response. Additionally, negative enrichment was observed in cell adhesion-related pathways. In contrast, we observed a significant positive enrichment in translation-related pathways, such as tRNA wobble modification and ribosome assembly, as well as in mitochondrial energy metabolism and cellular inflammatory responses. Furthermore, heatmaps were generated to display the differential expression of key molecules involved in the aforementioned functional pathways between tumor cells with high CTU2 expression and those with low CTU2 expression (**Figure 7D**). For instance, molecules associated with antigen presentation, such as those processed and presented by antigen-presenting HLA, were found to be expressed at lower levels in tumor cells with high CTU2 expression. In conclusion, the above results, from multiple perspectives, indicate the significant potential of CTU2 in tumors for immunotherapy response, particularly in immune evasion, suggesting its promising utility as a biomarker for cancer immunotherapy.

### 3.7 CTU2 functions as an oncogene across various cancer types

To anticipate the potential roles and underlying mechanisms of CTU2 in pan-cancer, GSEA was employed to enrich CTU2-associated Kyoto Encyclopedia of Genes and Genomes (KEGG) pathways and Gene Ontology (GO) biological processes. Numerous cancer-related pathways were notably enriched (**Supplementary Figure S8A**), including cell cycle (**Supplementary Figure S9A**), DNA replication (**Supplementary Figure S9B**), base excision repair (**Supplementary Figure S9C**), nucleotide excision repair (**Supplementary Figure S9D**), spliceosome (**Supplementary Figure S9E**), and proteasome (**Supplementary Figure S9F**), along with focal adhesion and cell adhesion molecules. In addition, pathways involved in protein folding, tRNA metabolic progress, and tRNA modification were also significantly enriched in this analysis, highlighting the significant role of CTU2 in tRNA physiological function and protein synthesis (**Supplementary Figure S8B**). While, the majority of cell-matrix adhesion-related genes were negatively correlated with CTU2, especially in CESC, DLBC, LGG, READ, and TGCT (**Supplementary Figure S8A**). The correlation analysis unveiled that CTU2 expression was additionally linked to various well-known oncogenes (**Supplementary Figure S8C**), including E2F transcription factor family members (**Supplementary Figures S9G, H**) and cell division cycle (CDC) protein (CCD45, CDC20) (**Supplementary Figures S9I, J**), and PLK1. Furthermore, the correlation analysis indicated that majority of genes linked to DNA replication and Base excision repair pathways exhibited positive correlations with CTU2 expression in KIRC (**Supplementary Figures S10A, B**) and LIHC (**Supplementary Figures S10C, D**). These findings suggest that targeting CTU2 and its associated pathways could be a viable strategy for developing new cancer therapies.

To further investigate the direct role of CTU2 in tumor cell function, we supplemented our analysis with data from the DepMap database. The DepMap database integrates data from thousands of cancer cell lines, known as the Cancer Cell Line Encyclopedia (CCLE), and conducts large-scale loss-of-function screens using CRISPR interference (CRISPRi) or RNA interference (RNAi) to evaluate gene essentiality. Specifically, when the loss or reduction of a gene significantly affects cell viability or fitness, the more negative the gene effect score, the stronger the gene dependency. As shown in **Supplementary Figure 11A**, knockdown or knockout of CTU2 impaired the proliferation of various cancer cell lines, with the gene effect scores being negative in nearly all of the cell lines, indicating a crucial gene dependency on CTU2 in the majority of cancer cells (**Supplementary Figure S11B**).

To further investigate the potential biological functions of CTU2 in pan-cancer, we examined whether cancer cell lines expressing high levels of CTU2 differ functionally from those with low levels. Functional enrichment analysis revealed a positive correlation between CTU2 expression and gene dependency in pathways involved in translation and tRNA aminoacylation (that is, higher CTU2 expression correlates with stronger dependency of these genes for cell survival) (**Supplementary Figures S11C, D**). This suggests that cancer cell lines with elevated CTU2 may regulate translation across multiple cancer types, which is consistent with the results shown in **Supplementary Figure 8**. Interestingly, we also observed a negative correlation between CTU2 expression and the gene dependency of canonical tumor suppressor genes, such as PTEN and RUNX3 (**Supplementary Figures S11C, D**), with these genes becoming less essential in CCLE-included cancer cell lines overexpressing CTU2.

### 3.8 CTU2 knockdown suppresses cell proliferation and migration

**Figure 8:**
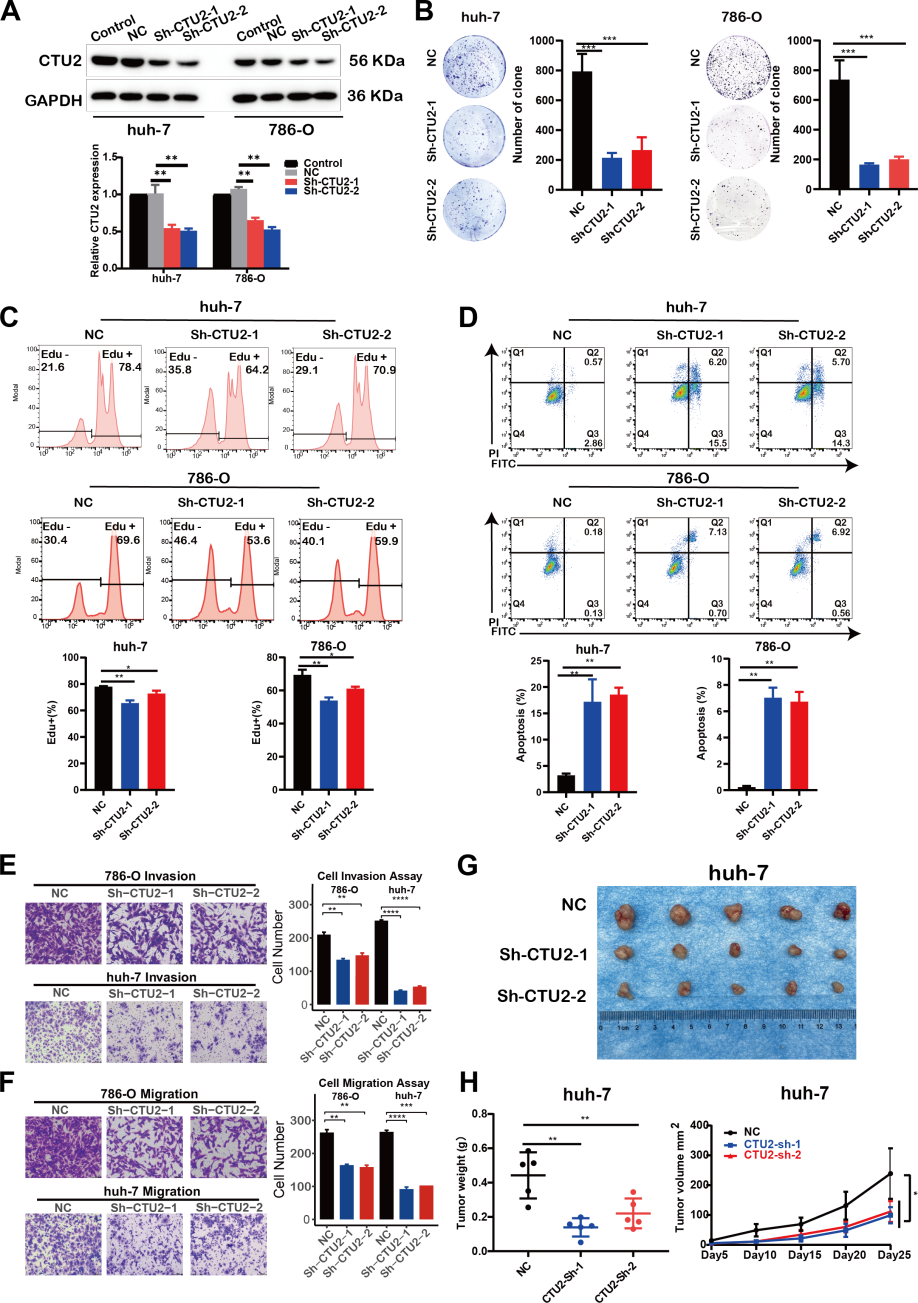
CTU2 knockdown impairs LIHC and KIRC progression *in vivo* and *in vitro*. **(A)** Western blot confirming CTU2 knockdown in huh-7 and 786-O. Control (untransfected wild-type cells), NC (lentiviral empty vector group), Sh-CTU2 (lentivirus-mediated CTU2 knockdown). The lower graph shows the grey value of CTU2 protein levels, normalized to the corresponding GAPDH levels. The experiment was independently repeated three times (**P < 0.01); **(B)** Colony-formation assay of CTU2 knockdown and control huh-7 and 786-O, representative images (left panel), and the quantitative analysis (right panel, ***P< 0.001); **(C)** EdU proliferation assay (upper) and the quantitative analysis (lower) of CTU2 knockdown and control cells (*P<0.05, **P < 0.01); **(D)** AnnexinV/PI apoptosis assay (upper) and the quantitative analysis (lower) of CTU2 knockdown and control cells (*P<0.05, **P < 0.01); **(E)** Matrigel invasion assay of CTU2 knockdown and control 786-O (upper) and huh-7 (lower) cell, representative images (left panel), and quantification analysis (right panel, **P < 0.01, ***P< 0.001, ****P< 0.0001); **(F)** Transwell cell migration analysis of CTU2 knockdown and control 786-O (upper) and huh-7 (lower) cell, representative images (left panel), and quantification analysis (right panel); **(G)** Representative picture of tumors in xenograft nude mice model subcutaneously implanted with CTU2 knockdown and control huh-7 cells; **(H)** Xenograft tumor weigh (left, n = 5, **P < 0.01) and xenograft tumor growth curve (right, n = 5, *P < 0.05).

To further validate the functional role of CTU2 predicted by multi-omics analyses in tumors, we constructed CTU2 stably knockdown cells using the LIHC cell line (huh-7) and the KIRC cell line (786-O), and the efficiency of CTU2 knockdown was confirmed by Western blot (**Figure 8A**). Clone formation assays showed that CTU2 knockdown significantly inhibited the clone formation of huh-7 and 786-O (**Figure 8B**). Flow cytometric analysis revealed that compared with the cells transfected with empty vector (shNC) in both cell types, inhibition of CTU2 expression reduced the number of EdU-positive S phase cells (**Figure 8C**) and increased the proportion of apoptotic cells (early apoptosis plus late apoptosis) (**Figure 8D**). Additionally, transwell migration and invasion assays indicated that CTU2 knockdown inhibited cell migration and invasion in both cell lines (**Figures 8E, F**). To go a step further, we performed subcutaneous tumor experiments by huh-7 cells to explore the effects of CTU2 on the tumorigenic ability *in vivo*. Consistent with the *in vivo* results, CTU2 silencing inhibited subcutaneous huh-7 xenograft growth in nude mice (**Figures 8G, H**). Altogether, results from *in vitro* and *in vivo* were consistent with the findings from prognostic analyses and gene set enrichment analysis, indicating that CTU2 may serve as an oncogene in cancer.

### 3.9 Drug sensitivity analysis identifies potential compounds targeting CTU2 in pan-cancer

**Figure 9:**
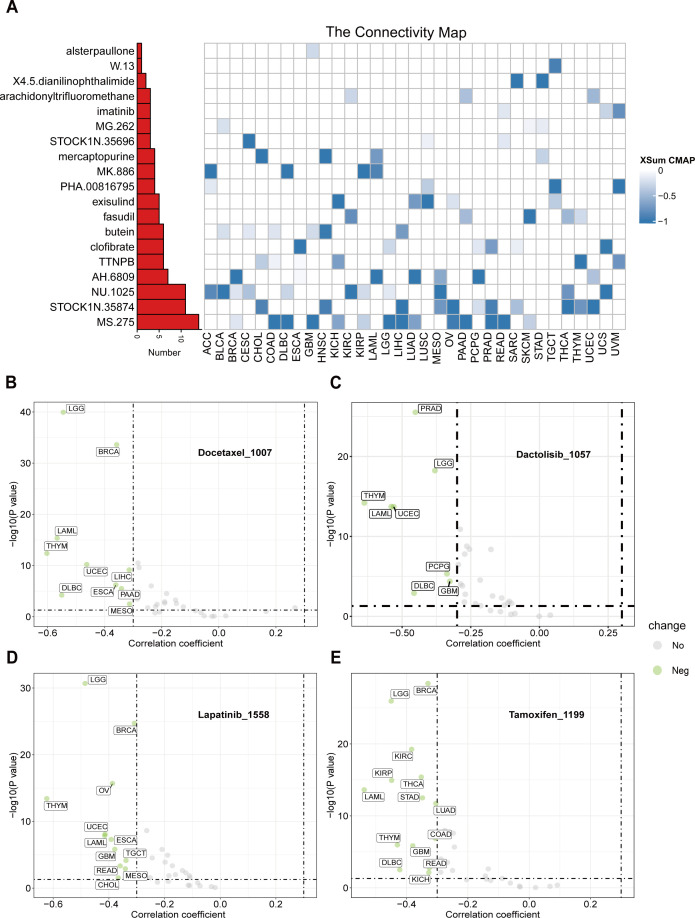
CTU2 is linked to the sensitivity of antitumor drugs across 33 cancer types. **(A)** A heatmap presentation shows the 19 candidate compounds that may target CTU2 based on the connectivity map analysis in 33 cancer types. The color codes from white to blue represent the XSum score from 0 to -1, respectively; Based on the ‘oncoPredict’ package, scatter plots present the Spearman correlation analysis results between CTU2 expression and drug sensitivity in **(B)** Docetaxel_1007, **(C)** Dactolisib_1057, **(D)** Lapatinib_1558, and **(E)** Tamoxifen_1199.

To identify potential therapeutic strategies targeting the tumor-promoting effects mediated by CTU2, we conducted a CMap analysis and developed a CTU2-related gene signature. This signature was created by selecting the top 150 significantly upregulated and 150 significantly downregulated genes from comparisons between CTU2-high and CTU2-low expressing patients across various cancer types. We employed the eXtreme-Sum (XSum) method, an optimized signature matching approach, to align the CTU2-related signature with CMap gene signatures. This analysis identified 1,288 compounds with similarity scores. Heatmap clustering analysis revealed 19 compounds with the top three lowest scores across 31 cancer types (**Figure 9A**). Notably, MS-275, STOCK1N.35874, and NU.1025 consistently exhibited significantly lower scores across multiple cancer types, suggesting their potential to counteract the pro-oncogenic effects of CTU2. Particularly, MS-275, a histone deacetylase (HDAC) inhibitor, targets HDAC enzymes and has shown anti-tumor effects in cancers such as leukemia, COAD, uveal melanoma, ESCA, BRCA, and HNSC. In 2024, after completing Phase III clinical trials (NCT03538171), it was approved for treating locally advanced or metastatic breast cancer, highlighting its potential in targeting CTU2-associated tumor progression (49). Additionally, using the ‘OncoPredict’ package and the GDSCv2 database, we assessed the sensitivity of 198 anti-tumor drugs (**Supplementary Table S5**). This analysis identified several drugs, such as Docetaxel_1007 (**Figure 9B**), Dactolisib_1057 (**Figure 9C**), Lapatinib_1558 (**Figure 9D**), and Tamoxifen_1199 (**Figure 9E**), with their sensitivity correlating with CTU2 expression levels, demonstrating a cancer-type-dependent response.

### 3.10 Copy Number Variation (CNV) and DNA methylation alterations of CTU2 across different human cancers

**Figure 10:**
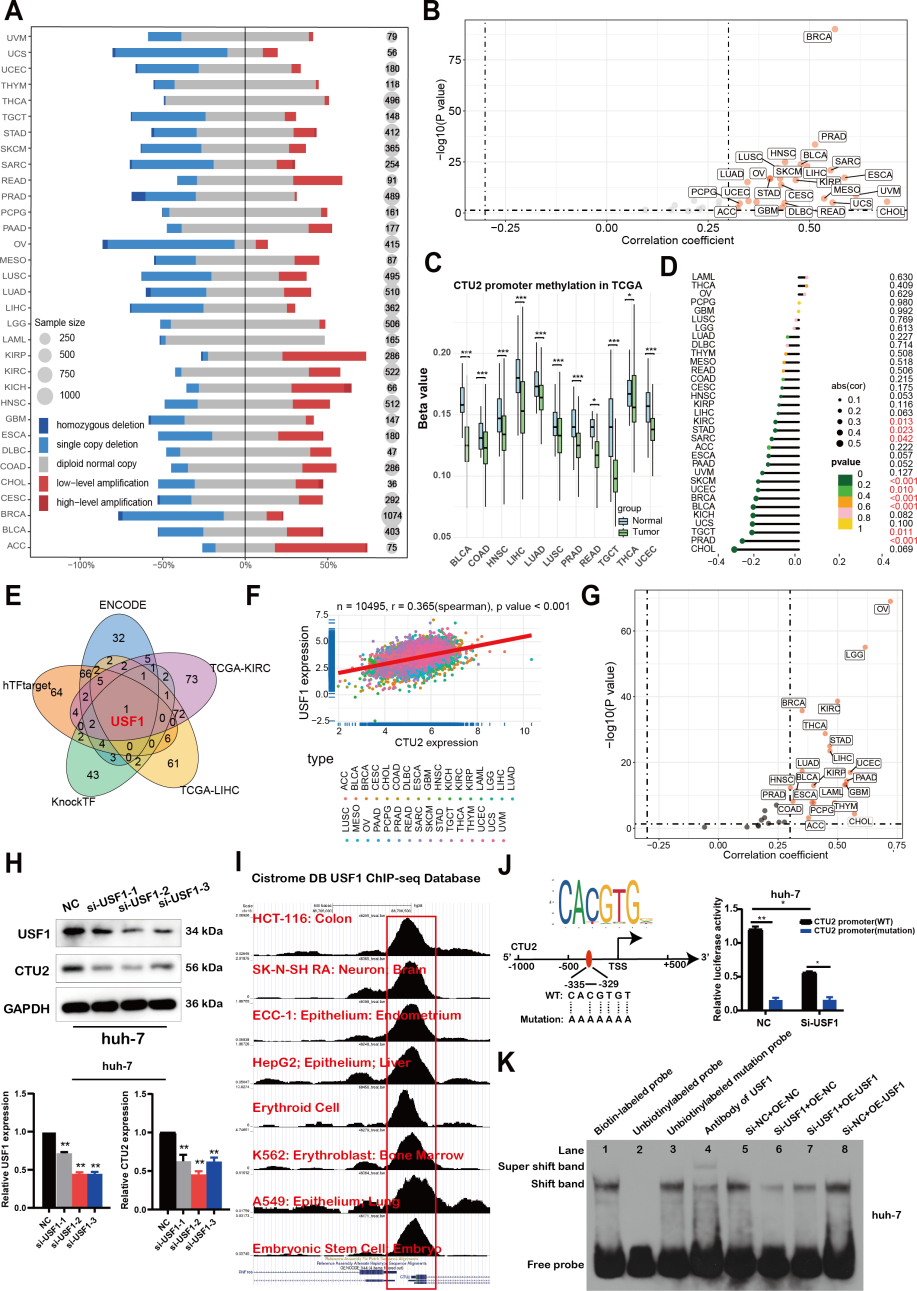
The mechanisms of upstream regulation of CTU2 expression in tumors. **(A)** DNA copy number variation analysis in 33 cancer types; **(B)** Scatter plot showing the results of Pearson correlation analysis in pan-cancer; **(C)** DNA methylation beta values ranging from 0 (unmethylated) to 1 (fully methylated) were determined by UALCAN; **(D)** Lollipop charts were used to visualize correlations between DNA methylation and mRNA expression of CTU2 (*P-value* < 0.05, marked in red font, shows statistical significance); **(E)** CTU2 upstream transcription factors prediction based on three web tools and correlation analysis; **(F, G)** Correlation analysis between CTU2 and USF1 expression in TCGA; **(H)** Western blot analysis confirmed USF1 knockdown and its effect on CTU2 expression in huh-7 cells. The lower graphs show the grey values of USF1 and CTU2 protein levels, normalized to the corresponding GAPDH levels. The experiment was independently repeated three times. The asterisk (*) indicates a statistically significant difference compared with NC, **P < 0.01; **(I)** ChIP-Seq data from the Cistrome Data Browser database to show the USF1 binding peaks of CTU2 promoter regions; **(J)** The cartoon shows the sequence logo of the USF1 potential binding site generated using JASPAR software (upper panel, http://jaspar.genereg.net/), with wild-type (WT) and mutated (Mutation) recognition sites of USF1 in the CTU2 promoter region depicted in the lower panel (left part). Luciferase assays demonstrated that USF1-mediated CTU2 promoter activity was significantly reduced following USF1 knockdown (right part); **(K)** EMSA analysis to evaluate the binding of the USF1 to the E-box motif in the CTU2 promoter under varying conditions of USF1 protein expression inhibition.

In order to uncover the mechanism underlying the elevated expression of CTU2, we conducted analyses on copy number variation of the CTU2 gene and DNA methylation alteration in the CTU2 promotor region. With regards to copy number variation, a higher prevalence of copy number gains was observed in CTU2 genes across various cancers such as ACC, KIRC, KIRP, and others (**Figure 10A**). Additionally, a significant positive correlation (Spearman r > 0.3; *P <* 0.05) was detected between CTU2 mRNA expression and copy number variation in the majority of tumor types (**Figure 10B**). We then investigated the differential promoter DNA methylation status of CTU2 between cancer and adjacent normal tissues by using UALCAN (**Figure 10C**). CTU2 had lower DNA methylation levels in BLCA, COAD, HNSC, LIHC, LUAD, LUSC, PRAD, READ, TGCT, THCA and UCEC tissues compared to adjacent normal tissues. To establish a connection between promoter DNA methylation levels and CTU2 expression, we conducted a correlation analysis between DNA methylation states and CTU2 expression (**Figure 10D**). A notable negative correlation was observed between DNA methylation and CTU2 expression in PRAD, TGCT, BLCA, BRCA, UCEC, SKCM, SARC, STAD, and KIRC (-0.3 < Spearman r < -0.1). Hence, the abnormal increase in CTU2 mRNA expression in certain cancers likely stems from both CNV alterations and reduced DNA methylation levels.

### 3.11 CTU2 is regulated by the transcription factor USF1

Finally, given the prognostic significance of CTU2, we performed promoter sequence analysis and used established transcription factor prediction tools, including ENCODE, hTF-target, and KnockTF, to identify potential upstream regulators of CTU2 expression. From these analyses and the correlation results of CTU2 in LIHC and KIRC datasets, we identified one common transcription factor, upstream transcription factor 1 (USF1) (**Figure 10E**). Correlation analysis showed a highly significant positive correlation between CTU2 and USF1 in the majority of TCGA datasets (**Figures 10F, G**). Consistent with these findings, USF1 knockdown led to a decrease in CTU2 expression in huh-7 (**Figure 10H**) and 786-O cell lines (**Supplementary Figure S12**). Further analysis of eight published USF1 ChIP-seq profiles available in the Cistrome Data Browser revealed high ChIP-seq binding peaks of USF1 at consistent locations within the CTU2 promoter regions (**Figure 10I**).

Additionally, USF1 DNA-binding motif prediction within the CTU2 promoter, conducted using JASPAR, confirmed the presence of conserved E-box binding sites for USF1 around the transcription start site (TSS). We constructed wild-type CTU2 promoter luciferase plasmids and plasmids containing mutations in the predicted USF1 binding sites (**Figure 10J**, left). Luciferase assays demonstrated that USF1 knockdown significantly reduced the relative luciferase activity of the CTU2-WT vector, while having minimal impact on the CTU2-mutated vector (**Figure 10J**, right). To further investigate the transcriptional regulation of CTU2 by USF1, we conducted an EMSA to assess binding of USF1 to the E-box motif in the CTU2 promoter (**Figure 10K**). Using a wild-type oligonucleotide probe and nuclear extracts from Huh-7 cells, we observed a reduction in protein-DNA binding upon USF1 knockdown (lane 6). However, overexpression of USF1 in knockdown cells partially restored the binding shift (lane 7). Furthermore, USF1 overexpression alone enhanced the protein-DNA binding shift compared to the empty vector control, indicating increased binding to the CTU2 promoter DNA. In summary, our findings suggest that CTU2 may be regulated by the transcription factor USF1.

## 4 Discussion

The traditional view posited that tRNAs were abundant, readily available, and merely passive participants in mRNA decoding and protein translation. However, accumulating evidence indicates that tRNA expression is cell-specific, tissue-specific, disease-specific, and temporally regulated (50, 51). The regulation of mRNA translation is a critical process in cancer initiation and progression, and aberrant modifications of tRNAs can affect translation in three primary ways: aberrant modifications in the anticodon that directly restrict or expand decoding functions; aberrant modifications in the tRNA body that alter its folding characteristics or structural stability; and aberrant modifications that alter charging specificity (52).

Recent studies have demonstrated that CTU2 is significantly overexpressed in breast cancer (16), drug-resistant melanoma (22), and activated T cells (53), where it drives mcm^5^s^2^U-modified tRNAs to decode U34 codons, selectively upregulating the translation efficiency of metastasis-related LEF1, glycolysis-related HIF1α, and stress-responsive transcription factor Atf4, all of which feature gene coding regions rich in U34 codons. It is evident that CTU2-mediated mcm^5^s^2^U modification primarily regulates tRNA decoding functions, thereby influencing the translation of functional genes (12). In contrast, recent studies on the highly discussed methylation modifications, such as m^6^A, m^5^C, and m^1^A, primarily occurring in messenger RNA (mRNA), microRNA (miRNA), and long non-coding RNA (lncRNA), mainly affect RNA stability, splicing, and decay, which is a form of regulation at the transcriptional level (54, 55). While tRNA also undergoes methylation modifications such as m^7^G and m^3^C, these are predominantly located in the tRNA body and similarly mainly influence tRNA stability (8). According to the central dogma of molecular biology, genetic information flows from DNA to RNA to protein, with proteins acting as the direct and final executors of gene function (56). However, therapeutic strategies targeting the tumor translation machinery remain scarce (57). Therefore, this study systematically analyzes the expression, prognostic relevance, and functions of CTU2 across various cancer types, aiming to provide a potential intervention strategy for tumors through CTU2-mediated tRNA mcm^5^s^2^U modification.

Changes in expression levels within tumor tissues are essential for genes to perform significant regulatory functions. Through analysis of TCGA data, we found that CTU2 expression varied significantly across various tumors compared to the corresponding paracancerous tissues. Subsequently, Clinicopathological staging analysis, OS analysis, and DSS analysis also revealed a close correlation between CTU2 expression and the clinical prognosis of various cancers, particularly in KIRC and LIHC. The drug sensitivity data from the GDSC database and DNA methylation data from cBioPortal and UALCAN further support the important role of CTU2 in various cancers.

The results of all the aforementioned analyses suggest that CTU2 is a critical diagnostic and therapeutic target for a variety of cancers. We believe that developing specific inhibitors or activators targeting CTU2 could significantly improve the disease progression and prognosis for cancer patients. Notably, in recent years, tRNA therapies have regained attention and achieved remarkable progress (58, 59). Therefore, developing tRNA-based therapies targeting the tRNAs modified by CTU2 may also be a viable approach. In addition, tumor immunotherapy also has been an effective treatment against tumors. We have been identifying biomarkers that activate the tumor immune response and facilitate immune evasion. To our excitement, pan-cancer analysis results have unveiled that CTU2 might play a pivotal role in the immune response across a spectrum of cancers. Chemokines, a group of relatively small molecular-weight secreted proteins, drive the movement and function of immune cells by interacting with chemokine receptors (60). The MHC, well-known for its role in antigen presentation and processing, is essential for initiating immune responses against a variety of human diseases (61). Our co-expression analysis has revealed a close association between CTU2 and the expression of these genes involved in chemokines, chemokine receptors, and MHC across different cancers, strongly suggesting that CTU2 could be indispensable for immunotherapy in diverse tumor types.

In terms of function and mechanism, GSEA revealed that CTU2 may contribute to numerous critical cancer-related pathways and biological processes. Specifically, CTU2 was found to have significant effects on the cell cycle and DNA replication. Combined with our analytical results, CTU2 exhibited notable regulatory roles in KIRC and LIHC, both in terms of differential expression analysis and prognosis. Therefore, we selected CTU2 for further investigation in KIRC and LIHC to validate our analytical findings. Experiments *in vitro* further confirmed that CTU2 promotes cancer behavior by enhancing cell proliferation and migration. Mechanistically, multi-omics analysis revealed that CTU2 upregulation is regulated by DNA copy number amplification and promoter methylation modifications. Notably, the transcription factor USF1 was identified as a regulator of CTU2 expression and has been confirmed to be an oncogene widely expressed in multiple cancer types (62–64).

## 5 Conclusions

In summary, this work demonstrated that high CTU2 expression in patients is significantly associated with poor prognosis and highlighted its potential as a biomarker for modulating immune cell infiltration, particularly in immune evasion processes, potentially influencing the response to immunotherapy in human cancers. Furthermore, CTU2-modified tRNA-Lys-TTT correlates with unfavorable outcomes across various tumor types. We validated its regulatory functions in KIRC and LIHC. Mechanistically, the amplification of copy number variation, hypomethylation of the promoter, and transcriptional regulation by USF1 may drive CTU2 expression in tumors. Overall, this study provided a comprehensive overview of genetic landscape of CTU2 across cancer types, providing new insights and support for the role of tRNA modification enzymes in cancer therapy.

## Data Availability

The original contributions presented in the study are included in the article/**Supplementary Material**. Further inquiries can be directed to the corresponding authors.
