# Supplementary material for: Pan-cancer analysis identifies tRNA modification enzyme CTU2 as a novel tumor biomarker and its role in immune microenvironment
Source: Front Immunol. 2025 May 1;16:1547794. doi: 10.3389/fimmu.2025.1547794 (PMC12078242; doi:10.3389/fimmu.2025.1547794)
Supplement: Supplementary file 6 [file SupplementaryFile1.docx]

**
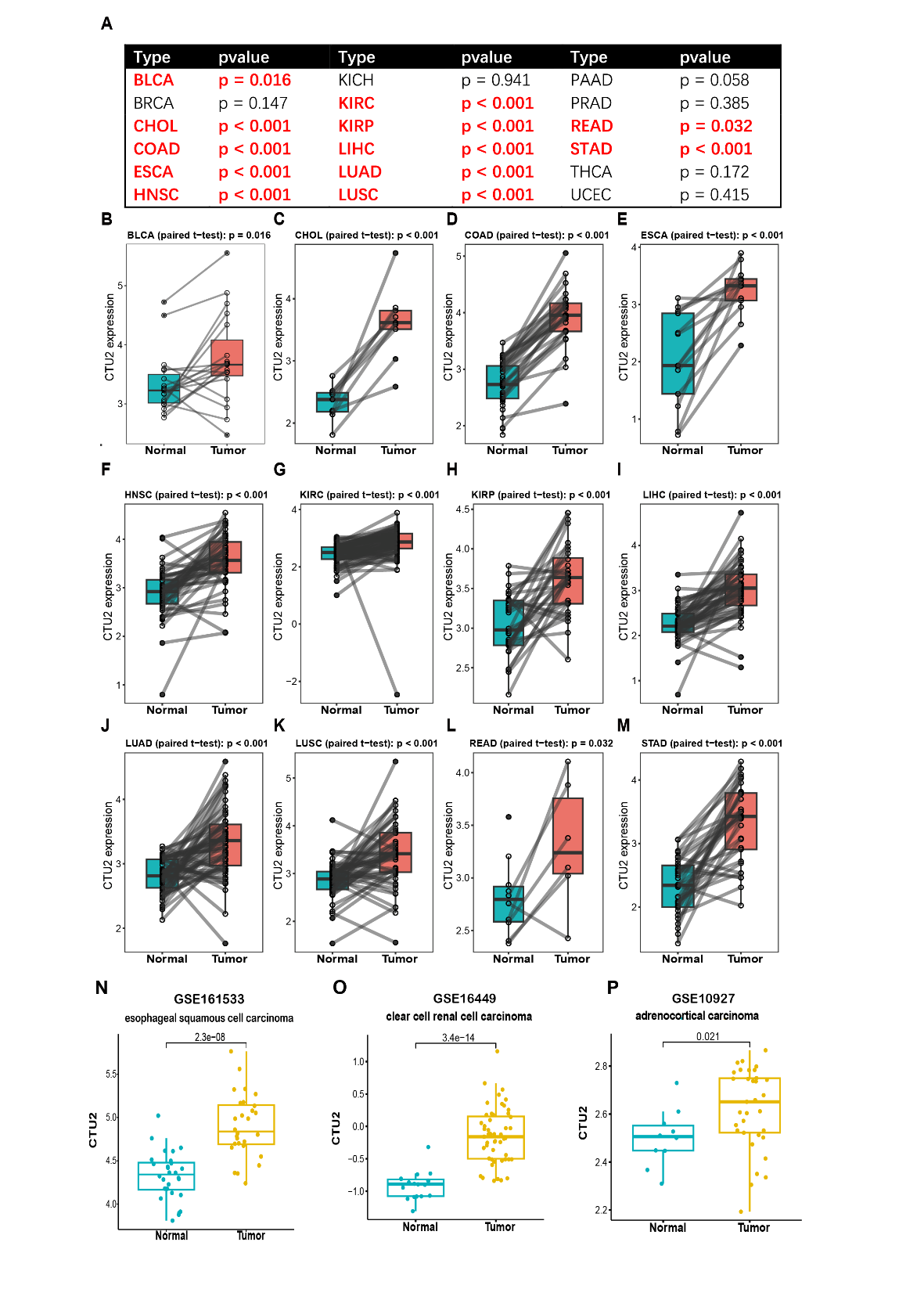
**

**Figure S1. CTU2 is upregulated in multiple cancer types.** (A) Overview of the results of paired Student’s t-test in 18 cancer types; (B-M) Results of paired Student’s t-test comparing CTU2 expression between tumor and adjacent normal tissues for a single cancer type in TCGA datasets; (N-P) Differential CTU2 mRNA expression in different cancer GEO datasets.


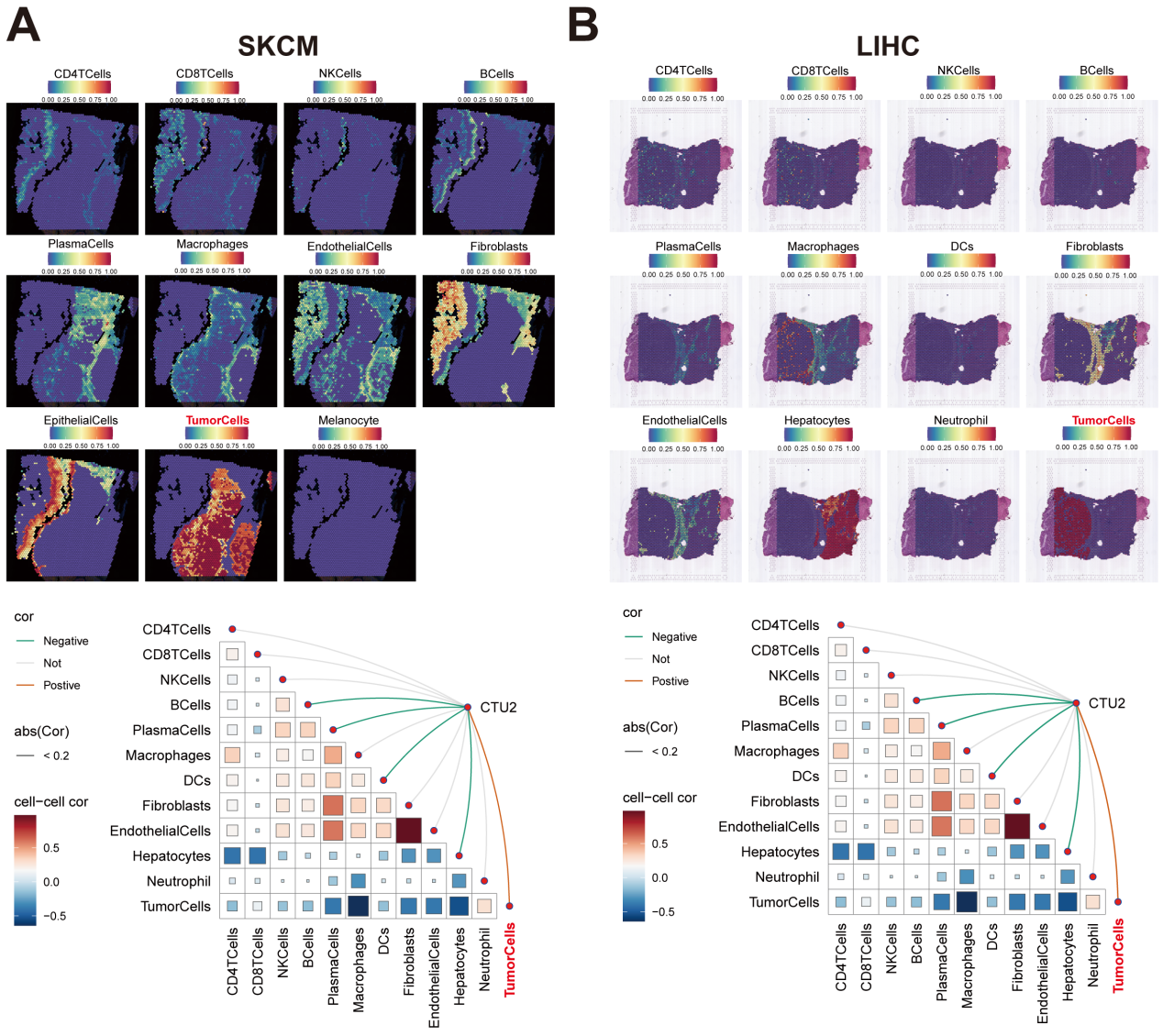


**Figure S2. Figure 3. Spatial transcriptomics of CTU2 expression in SKCM and LIHC.** Spatial transcriptomics deconvolution maps (upper panel) visualize the cell localization in SKCM (A) and LIHC (B). The panel below represents the Correlation Analysis between cell types located at different spatial locations and CTU2 expression levels.


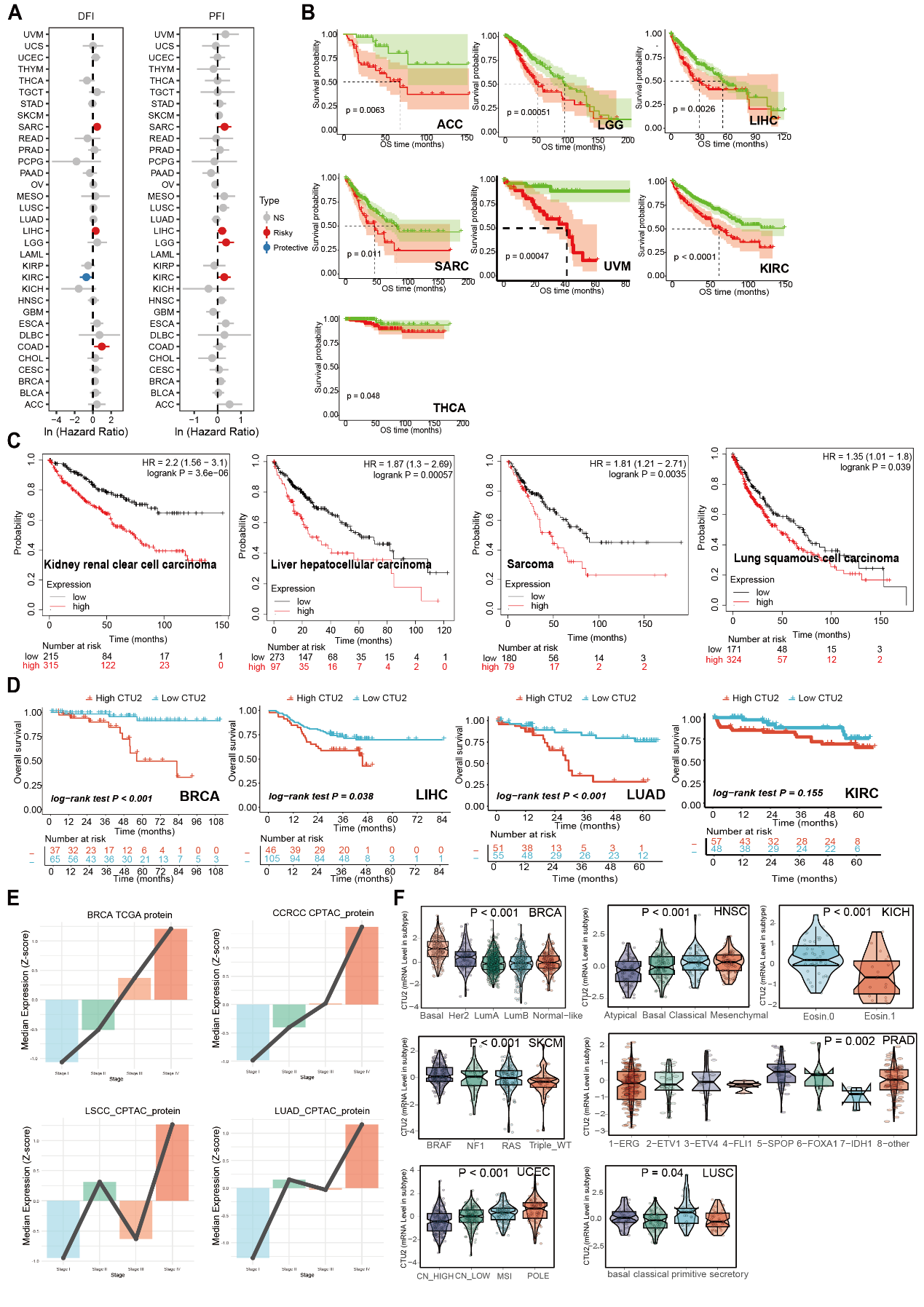


**Figure S3. Survival analysis of CTU2 in pan-cancer.** (A) DFI and PFI of CTU2 expression in 33 TCGA cancer types; (B) Kaplan-Meier survival analysis of OS for CTU2 mRNA expression in multiple cancer types from TCGA; (C) Kaplan-Meier analysis of OS for CTU2 mRNA expression in various cancer types based on the KM-plotter online tool; (D) Kaplan-Meier survival analysis of OS for CTU2 protein expression in different cancer types from CPTAC; (E) Variations in CTU2 protein expression across different stages of different tumors; (F) CTU2 mRNA expression in different molecular subtypes of TCGA tumors.

**
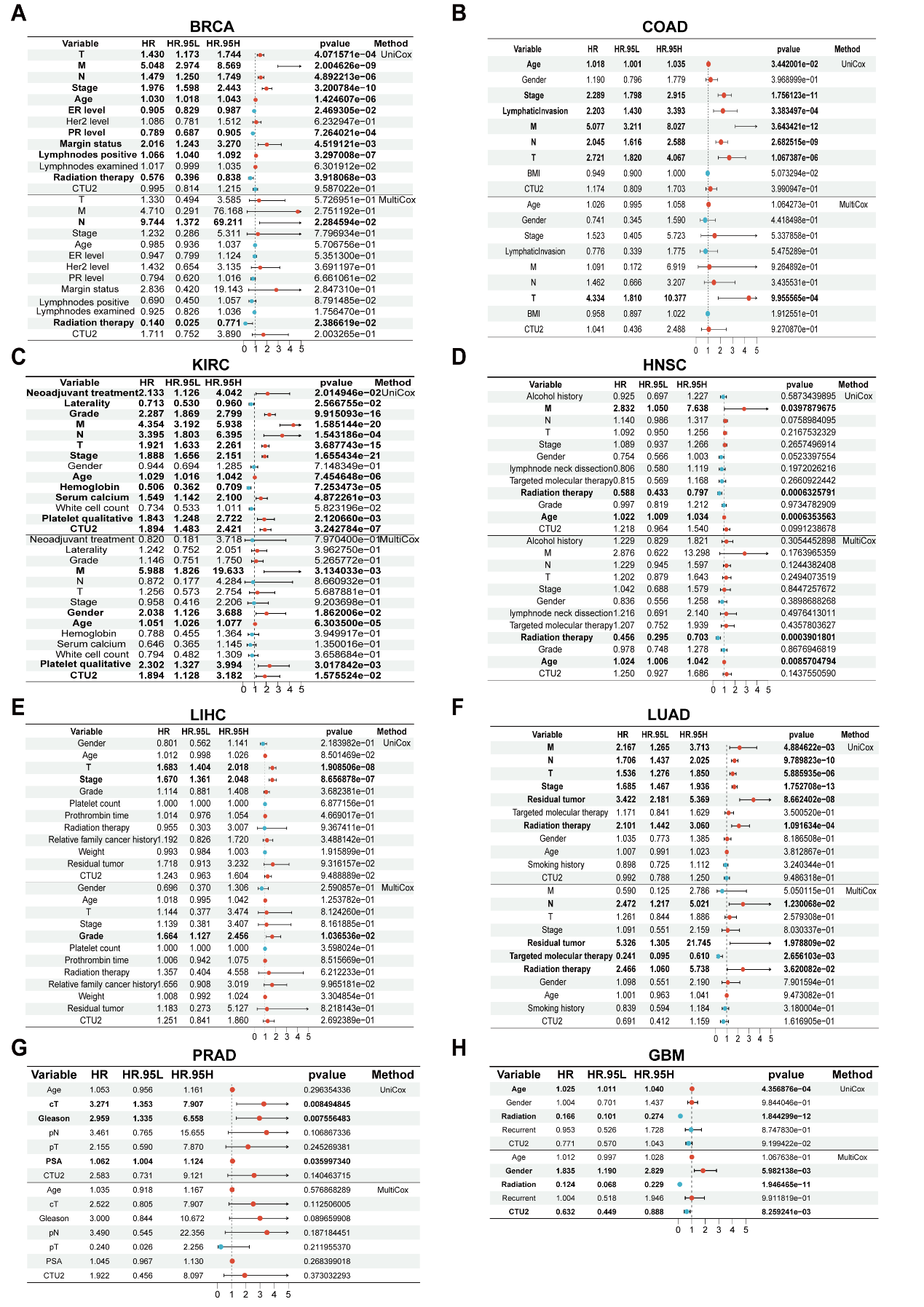
**

**Figure S4. Independent prognostic analysis of CTU2 expression and tumor survival.** (A-H) The analysis was conducted to determine whether the association between CTU2 expression and tumor survival is independent of traditional clinical variables in various cancer types. Above the horizontal line is the Univariate Cox survival analysis, and below is the Multivariate Cox survival analysis. Relative risks are described using hazard ratios (HR) and 95% confidence intervals (CI). HR greater than 1 means that it is a risk factor, and vice versa is a protective factor. *P-value* <0.05 is considered statistically significant and the characters are bolded.

**
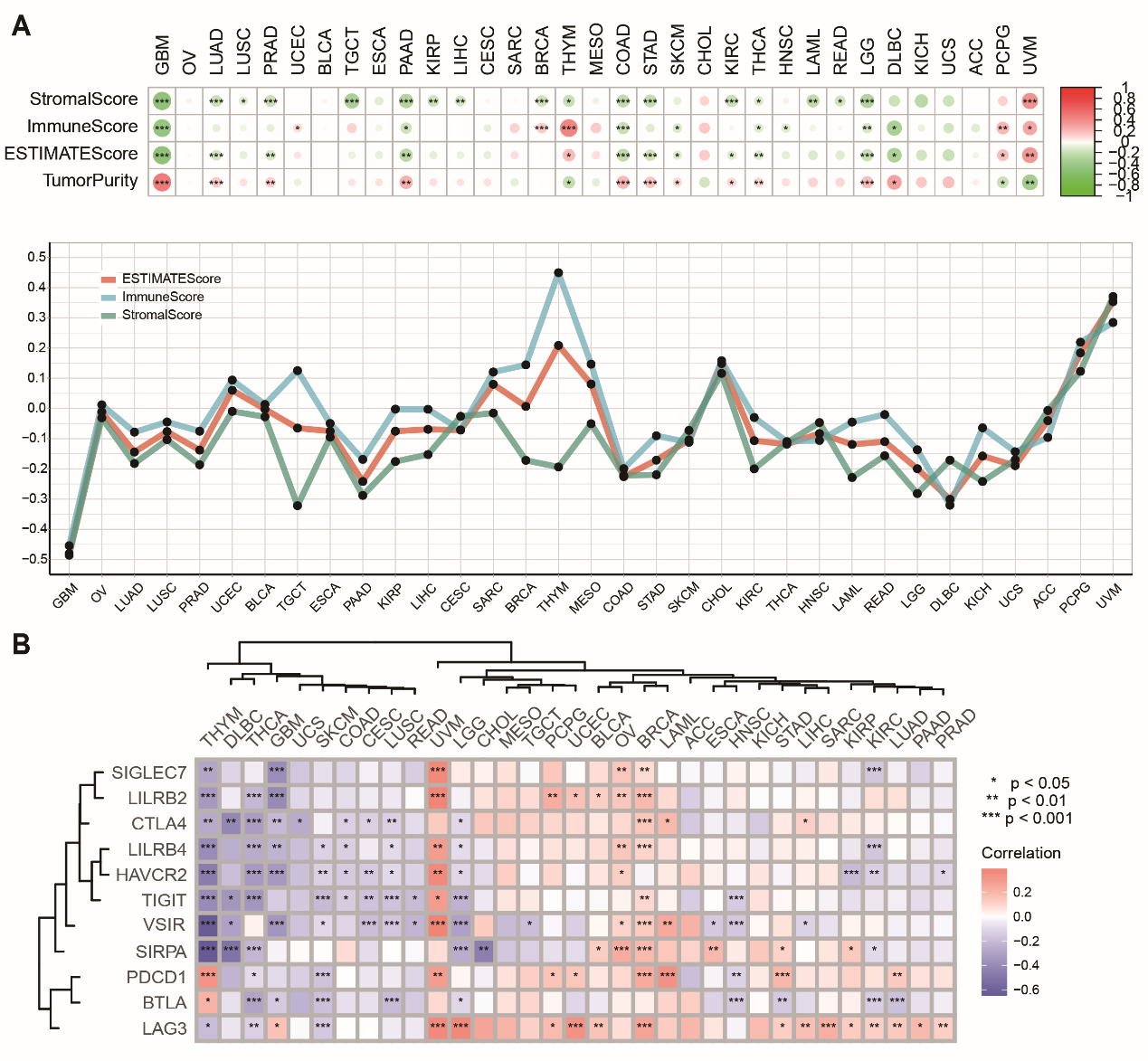
**

**Figure S5. Correlation Analysis of CTU2 with Immune Scores, checkpoint genes, and genomic instability markers.** (A) Correlation between CTU2 expression and ESTIMATE, Immune, and Stromal scores. Color ranging from green to red represents the correlation coefficient; (B) Heatmap shows the correlation between the expression of CTU2 and 11 immune checkpoint genes, *P-*value < 0.05 threshold.

**
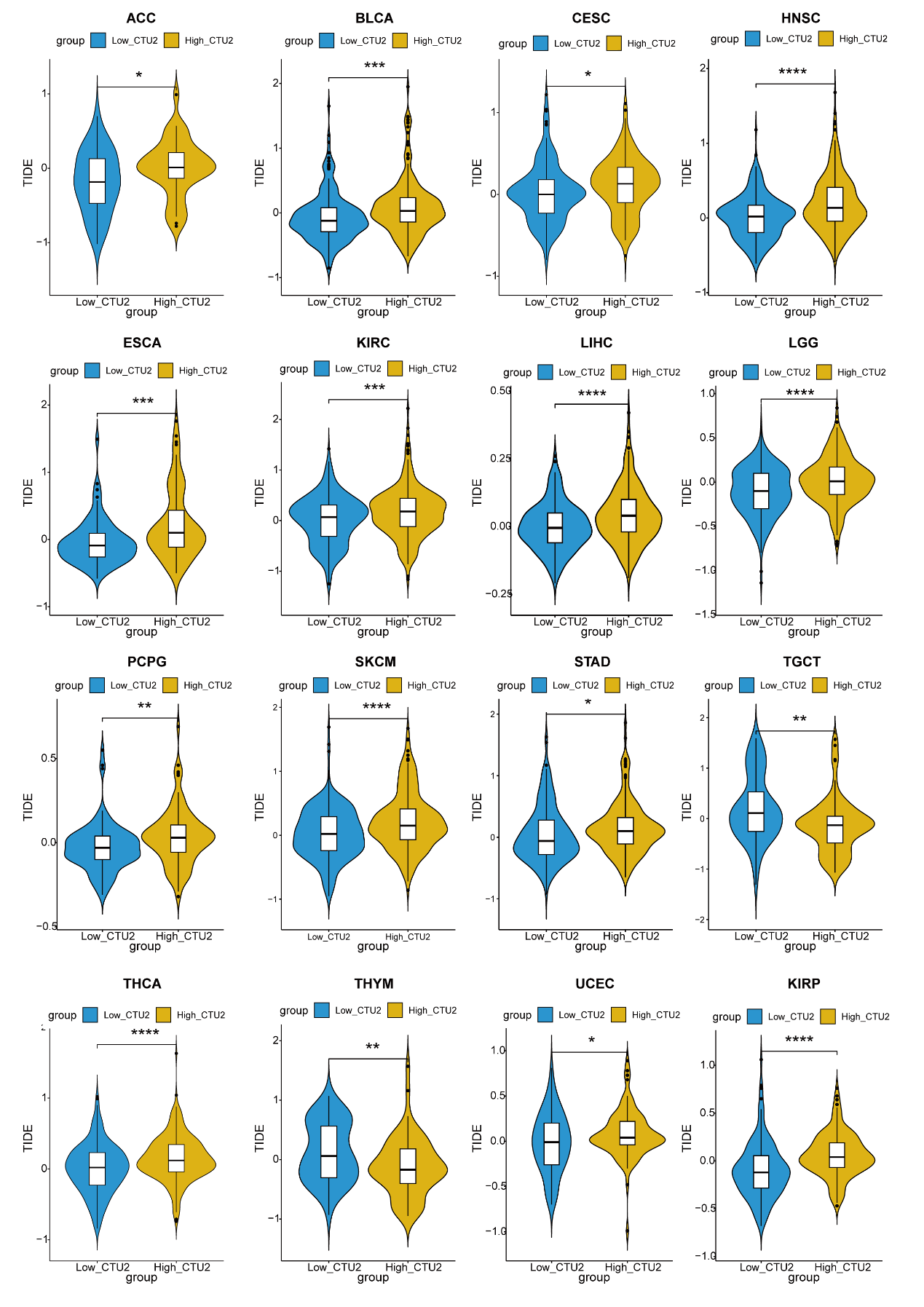
**

**Figure S6. The distribution of TIDE scores across CTU2 high and low expression groups in various tumors.** CTU2-high and CTU2-low is distinguished by the median.


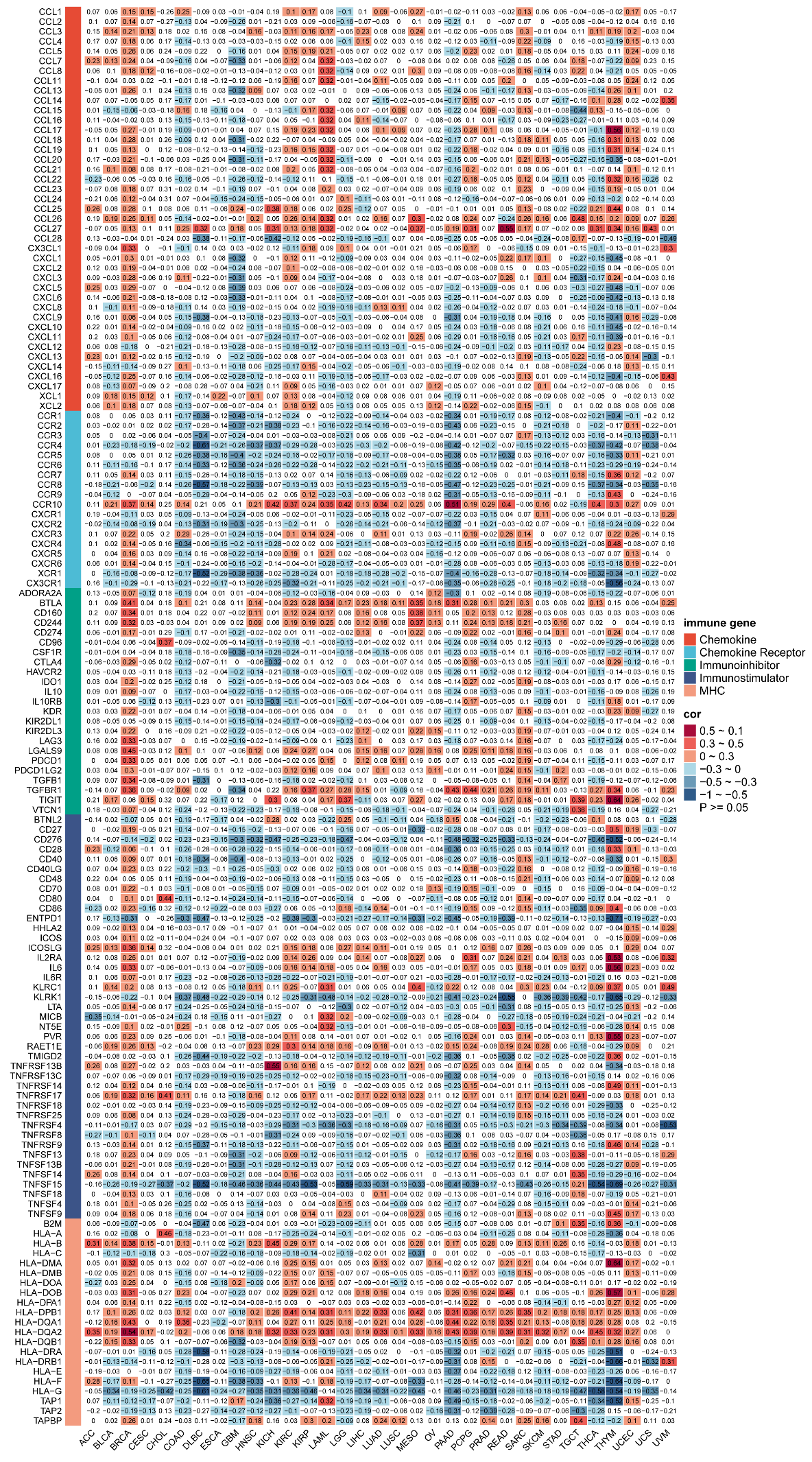


**Figure S7. Analysis of the correlation of the expression levels of CTU2 and immunomodulatory molecules in multiple cancer types.**


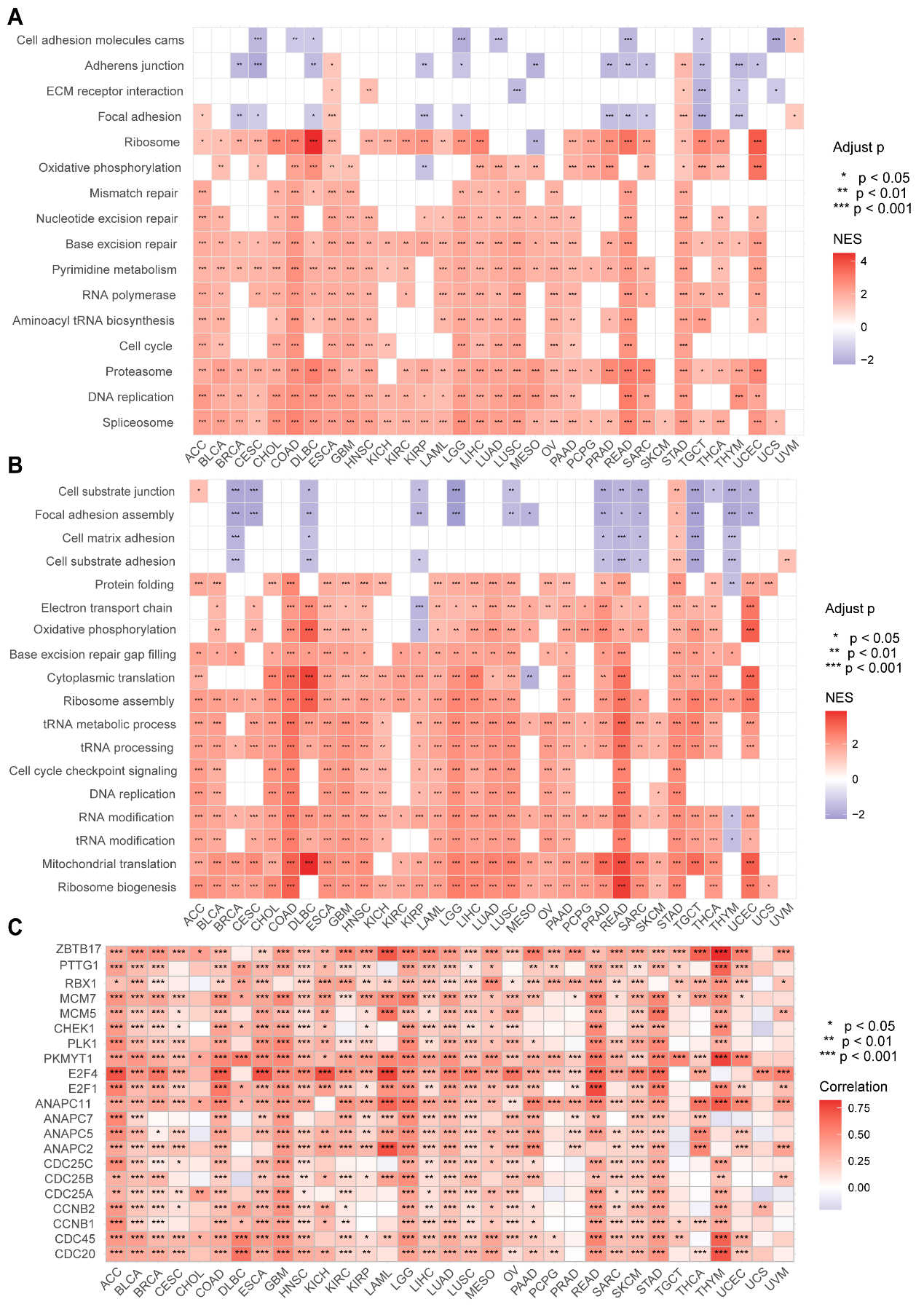


**Figure S8. CTU2 is associated with cancer-related pathways and biological processes.** (A) A heatmap displays KEGG pathways linked to CTU2 based on GSEA, with colors ranging from blue to red indicating the NES value; (B) Another heatmap illustrates GO-biological processes associated with CTU2 identified through GSEA, with colors ranging from blue to red representing the NES value; (C) A heatmap showcases cell cycle genes correlated with CTU2 based on correlation analysis, with colors ranging from blue to red representing the correlation coefficient.

**
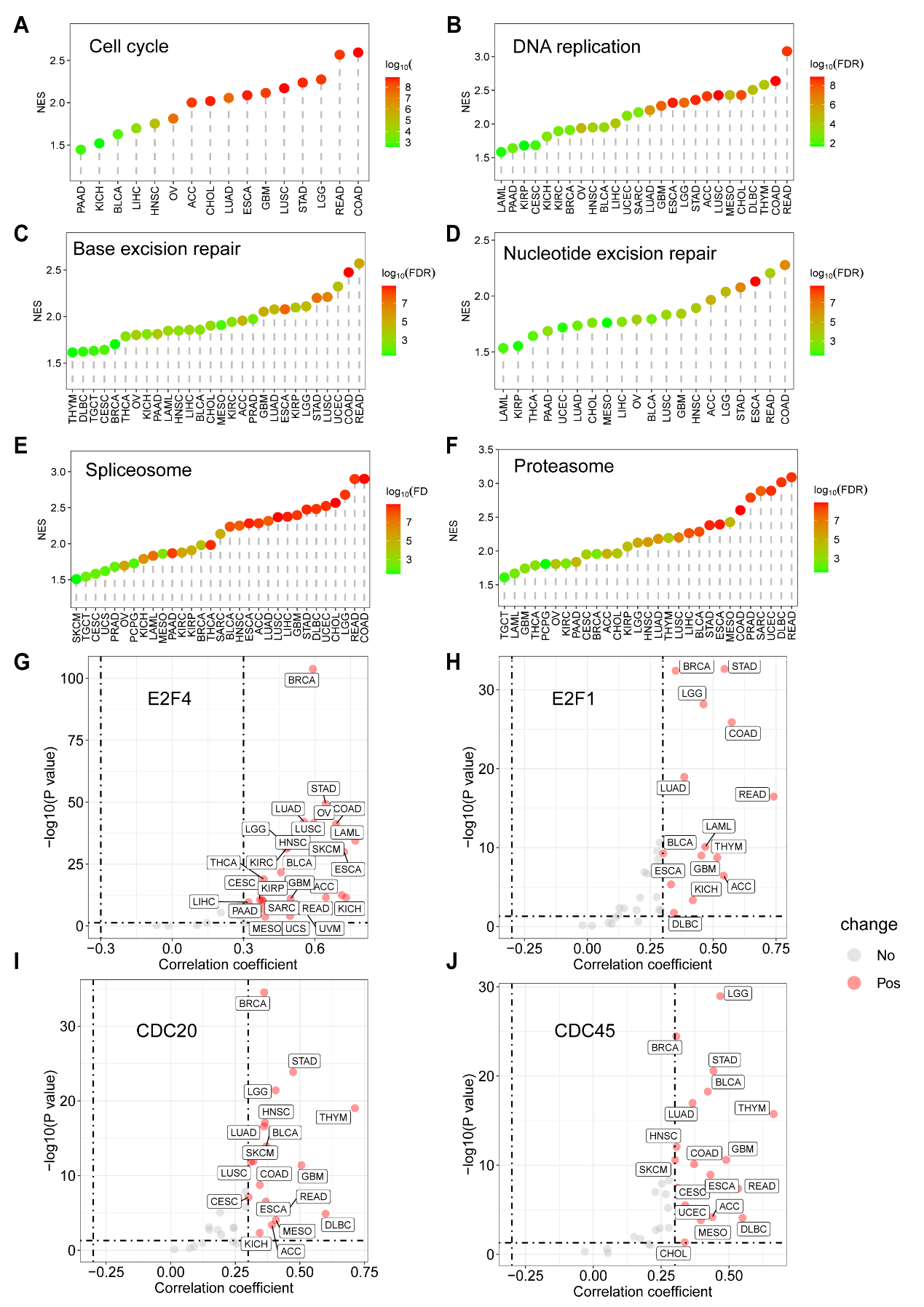
**

**Figure S9. CTU2 expression is correlated with several vital biological processes and oncogenes.** (A-F) Lollipop plots show the correlation between CTU2 expression and multiple biological processes in pan-cancer; (G-J) Scatter plots show the correlation between the expression of CTU2 and 4 oncogenes.


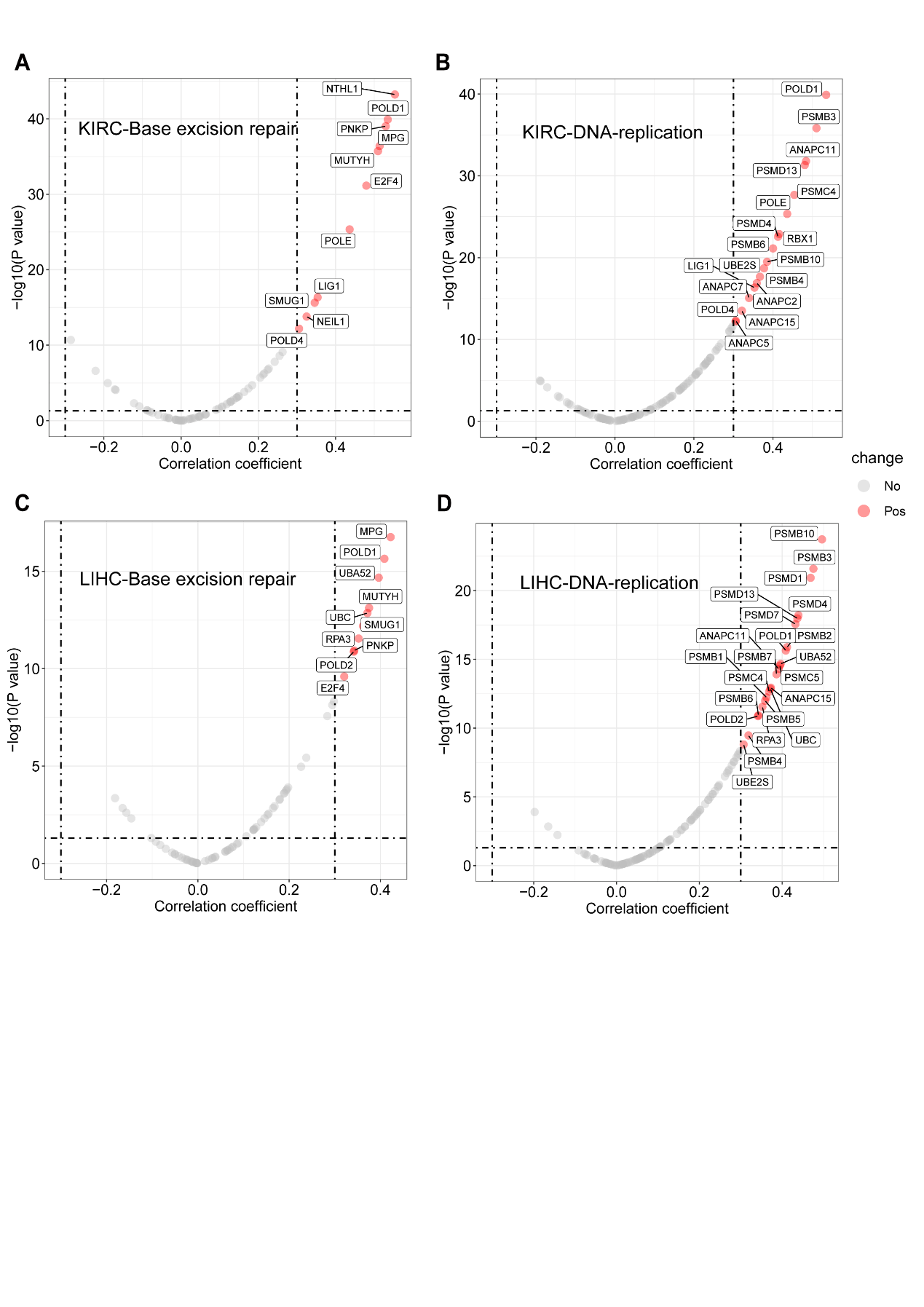


**Figure S10. CTU2 expression is correlated with several pathways-related genes.** Scatter plots show the correlation between the expression of CTU2 and base excision repair-associated genes in TCGA (A) KIRC and (C) LIHC datasets. Scatter plots show the correlation between the expression of CTU2 and DNA repair-associated genes in TCGA (B) KIRC and (D) LIHC.


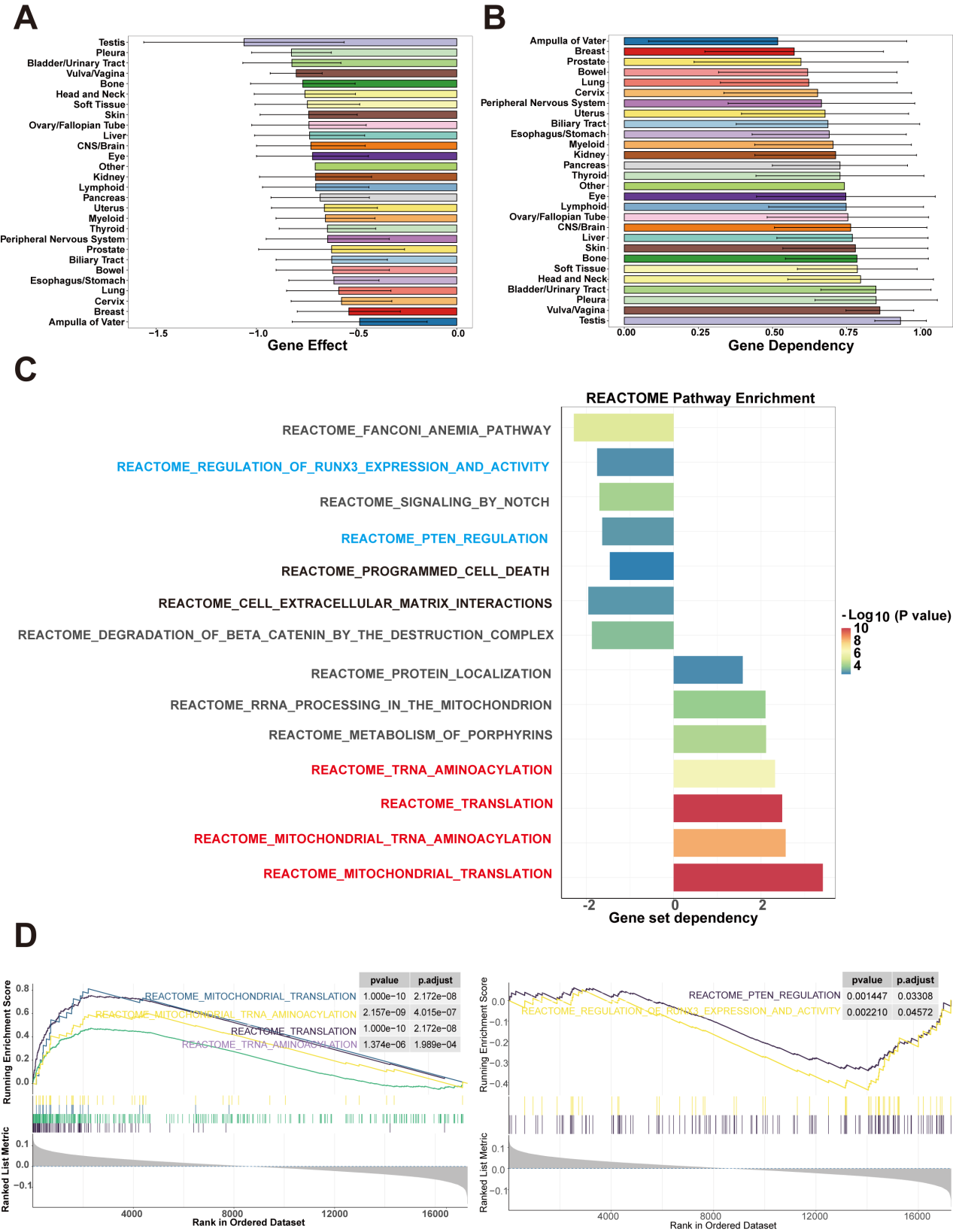


**Figure S11. DepMap Analysis Reveals CTU2's Association with Cancer-Related and Translation-Related Pathways.** (A) The gene effect score of CTU2 across pan-cancer cell lines in the DepMap database; (B) The gene dependency of CTU2 across pan-cancer cell lines in the DepMap database, in contrast to the gene effect score; (C) REACTOME gene set enrichment for genes that become less essential with increasing CTU2 mRNA levels in a DepMap analysis; (D) CTU2 is associated with the positively gene-dependent pathway (left) and the negatively gene-dependent pathway (right).


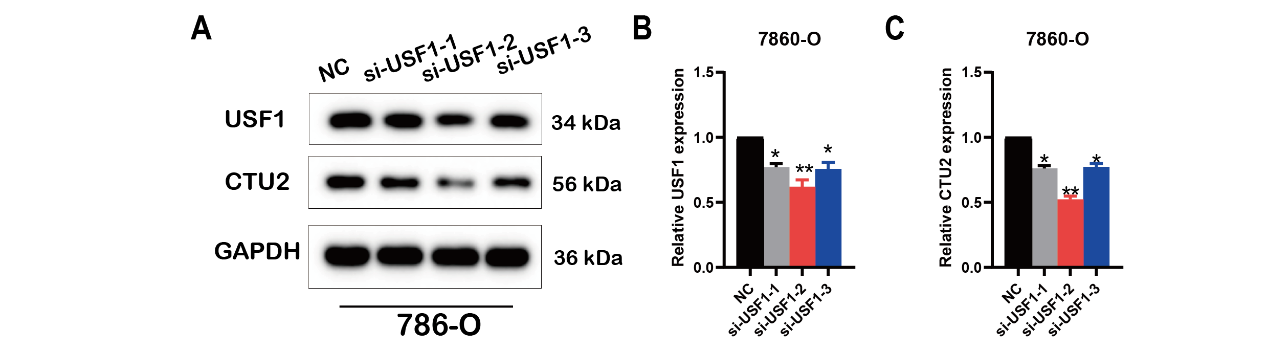


**Figure S12. Regulatory Effect of USF1 on CTU2 Expression.** (A) Western blot analysis confirmed USF1 knockdown and its effect on CTU2 expression in 786-O cells. The graphs show the grey values of USF1 (B) and CTU2 (C) protein levels, normalized to the corresponding GAPDH levels. The experiment was independently repeated three times. The asterisk (*) indicates a statistically significant difference compared with NC, **P* < 0.05, ***P* < 0.01.
